# Identification of the Elusive Pyruvate Reductase of *Chlamydomonas reinhardtii* Chloroplasts

**DOI:** 10.1093/pcp/pcv167

**Published:** 2015-11-15

**Authors:** Steven J. Burgess, Hussein Taha, Justin A. Yeoman, Oksana Iamshanova, Kher Xing Chan, Marko Boehm, Volker Behrends, Jacob G. Bundy, Wojciech Bialek, James W. Murray, Peter J. Nixon

**Affiliations:** ^1^Department of Life Sciences, Sir Ernst Chain Building-Wolfson Laboratories, Imperial College London, S. Kensington Campus, London SW7 2AZ, UK; ^2^Department of Plant Sciences, University of Cambridge, Downing Street, Cambridge CB2 3EA, UK; ^3^Department of Biomolecular Medicine, Sir Alexander Fleming Building, Imperial College London, S. Kensington Campus, London SW7 2AZ, UK; ^4^These authors contributed equally to this work; ^5^Present address: Faculty of Science, Universiti Brunei Darussalam, Jalan Tungku Link, BE1410, Brunei Darussalam

**Keywords:** Biohydrogen, *Chlamydomonas*, Fermentation, Lactate, LDH

## Abstract

Under anoxic conditions the green alga *Chlamydomonas reinhardtii* activates various fermentation pathways leading to the creation of formate, acetate, ethanol and small amounts of other metabolites including d-lactate and hydrogen. Progress has been made in identifying the enzymes involved in these pathways and their subcellular locations; however, the identity of the enzyme involved in reducing pyruvate to d-lactate has remained unclear. Based on sequence comparisons, enzyme activity measurements, X-ray crystallography, biochemical fractionation and analysis of knock-down mutants, we conclude that pyruvate reduction in the chloroplast is catalyzed by a tetrameric NAD^+^-dependent d-lactate dehydrogenase encoded by Cre07.g324550. Its expression during aerobic growth supports a possible function as a ‘lactate valve’ for the export of lactate to the mitochondrion for oxidation by cytochrome-dependent d-lactate dehydrogenases and by glycolate dehydrogenase. We also present a revised spatial model of fermentation based on our immunochemical detection of the likely pyruvate decarboxylase, PDC3, in the cytoplasm.

## Introduction

The unicellular green alga *Chlamydomonas reinhardtii*, which is found in fresh water and soil, has a versatile metabolism allowing it to acclimate to a wide range of environments and stresses (Catalanotti et al. 2013).

In the light, ATP is generated by photophosphorylation in the chloroplast, and under aerobic conditions by oxidative phosphorylation in the mitochondrion using oxygen as the final electron acceptor. When cells encounter anoxic conditions, the tricarboxylic acid (TCA) cycle shuts down, necessitating ATP generation by glycolysis, with NAD^+^ regenerated through a number of different fermentative pathways, leading to the excretion of formate, ethanol and acetate (Gfeller and Gibbs 1984), with hydrogen (Gfeller and Gibbs 1984, Kreuzberg 1984), glycerol (Klein and Betz 1978, Gfeller and Gibbs 1984, Meuser et al. 2009), succinate (Dubini et al. 2009), lactate (Gfeller and Gibbs 1984, Husic and Tolbert 1985) and malic acid (Mus et al. 2007) also produced, but at lower levels. This is accompanied by a concomitant reorganization in nitrogen metabolism, and activation of glyoxylate and reductive pentose phosphate pathways (Subramanian et al. 2014).

Understanding the fermentative pathways in *C. reinhardtii* has attracted interest because of the potential in using this and related algae for producing biohydrogen (Kruse et al. 2005), and the possibility that knocking out competing fermentation pathways might be one route to improve the yields of hydrogen.

Hydrogen is produced by the combined activity of two chloroplast-localized [Fe–Fe]-hydrogenases (HYDA1 and HYDA2) (Happe and Naber 1993, Forestier et al. 2003, Meuser et al. 2012) with electrons derived from reduced ferredoxin (PETF) (Winkler et al. 2009) produced by pyruvate:ferredoxin oxidoreductase (PFOR) and by PSI in the light (Noth et al. 2013, van Lis et al. 2013). Formate is produced by pyruvate formate lyase (PFL1) (Hemschemeier et al. 2008, Phillips et al. 2011, Burgess et al. 2012, Catalanotti et al. 2012), ethanol by a bifunctional acetaldehyde/alcohol dehydrogenase (ADH1) (Atteia et al. 2003, Magneschi et al. 2012) and acetate through the co-operation of phosphate acetyl transferases (PATs) and acetate kinases (ACKs), although alternative pathways also exist (Yang et al. 2014). Additionally there are three pyruvate decarboxylase sequences annotated in the genome. Two are likely to be E1 α components (EC 1.2.4.1; PDC1 and PDC2) of distinct mitochondrial (mtPDH) and plastid isoforms (plPDH) of the pyruvate dehydrogenase complex (PDH)—although this has yet to be verified (Supplementary Figs. S1–S3; Supplementary Tables S1, S2) (Burgess, 2011, Shtaida et al. 2014). The third, PDC3 (EC 4.1.1.1), is a likely pyruvate decarboxylase (Supplementary Figs. S4, S5) which could provide an additional route for pyruvate breakdown during anoxia, ultimately leading to the production of ethanol in concert with an ADH (Grossman et al. 2007). However, PDC3 remains uncharacterized and its subcellular location unknown.

Eliminating the main fermentation pathways has had unanticipated knock-on effects on metabolism, including the enhanced excretion of d-lactate in the case of *pfl1* and *adh1* mutants (Philipps et al. 2011, Catalanotti et al. 2012, Magneschi et al. 2012). An enzyme activity catalyzing the reduction of pyruvate to d-lactate was first investigated in *C. reinhardtii* by Husic and Tolbert (1985), but the pyruvate reductase was only partially purified and its identity has since remained unclear. Here we exploit the genome sequence of *C. reinhardtii* (Merchant et al. 2007) to identify this enzyme and to begin to address its physiological role through the generation of knock-down mutants. Further, we build on previous experiments attempting to define the subcellular location of fermentative pathways in *C. reinhardtii* (Kreuzberg et al. 1987, Terashima et al. 2010) to include an immunochemical analysis of the cytoplasm. Our data allow us to provide a refined description of pyruvate metabolism during anoxia.

## Results

### In silico identification of potential d-lactate dehydrogenase (d-LDH) enzymes

Unlike plants, *C. reinhardtii* has been shown both to produce (Husic and Tolbert 1985) and oxidize d-lactate (Husic and Tolbert 1987). To identify potential d-LDHs in *C. reinhardtii*, the genome sequence was searched with sequences of well-characterized NAD^+^-dependent d-LDH enzymes (d-nLDH; EC 1.1.1.28) from other species. Six related sequences were identified: Cre07.g324550, Cre01.g019100, Cre16.g689700, Cre07.g344550, Cre07.g344400 and Cre02.g087300 (Supplementary Fig. S6). Of these, the most likely d-nLDH is Cre07.g324550, which shows 30–33% protein sequence identity to known d-nLDH sequences and possesses the NAD^+^-binding domain (GXGX_2_GX_17_D) (Wierenga et al. 1986), and the conserved histidine (for catalysis), arginine (for substrate binding) and glutamic acid residues (for modulating pH dependence) (Lapierre et al*.* 1999) characteristic of such enzymes (Supplementary Fig. S7).

Sequence comparisons also revealed two potential NAD^+^-independent (d-iLDH; EC 1.1.2.4) enzymes: Cre10.g434900 and Cre08.g370550, which are respectively homologous to *Saccharomyces cerevisiae* DLD1 and DLD2/DLD3 (Supplementary Figs. S8, S9). The characterized DLD1s from *S. cerevisiae* and *Kluyveromyces lactis* have been shown to possess d-LDH activity (Lodi and Ferrero 1993, Lodi et al. 1994). Although DLD2 and DLD3 from *S. cerevisiae* also possess d-LDH activity (Chelstowska et al. 1999), the *Arabidopsis thaliana* ortholog was characterized as a highly specific d-2-hydroxyglutarate dehydrogenase (Engqvist et al. 2009), emphasizing the requirement for further characterization of the *C. reinhardtii* orthologs. However, irrespective of potential substrate specificities, this class of enzyme (EC 1.1.2.4) acts principally in the direction of pyruvate synthesis (Engqvist et al. 2009), so Cre10.g434900 and Cre08.g370550 are unlikely candidates for the pyruvate reductase involved in fermentation, and were not characterized further.

Close homologs of the Cyt-dependent (l-LDHc; EC 1.1.2.3) and NAD^+^-dependent (l-nLDH; EC 1.1.1.27) l-LDHs found in plants were not detected*.* However, more recent work has revealed that the annotated glycolate oxidase of *C. reinhardtii* possesses l-lactate oxidase activity, so other routes for synthesizing l-lactate cannot yet be excluded (Hackenberg et al. 2011).

### Cre07.g324550 functions as a pyruvate reductase

To examine the enzymatic function of Cre07.g324550, a C-terminal His-tagged derivative was expressed in *Escherichia coli* using a codon-optimized synthetic gene. As the gene product was predicted by ChloroP (Emanuelsson et al. 1999, Emanuelsson et al. 2000) to be targeted to the chloroplast, the putative transit peptide of 44 residues was removed and only residues 45–421 were expressed. The 40 kDa His-tagged derivative was expressed as a soluble protein that could be isolated by Ni^2+^ affinity chromatography (Supplementary Fig. S10A). The mass of the native species was determined to be approximately 200 kDa by size-exclusion chromatography, indicating the formation of an oligomer (Supplementary Fig. S10B, C). As expected from sequence comparisons, enzyme assays confirmed that the protein was a d-LDH with a preference for the reduction of pyruvate to d-lactate and a specificity for NADH (Table 1). The enzyme displayed Michaelis–Menten kinetics under the conditions examined (Supplementary Fig. S11) giving a *K*_M_ value in good agreement with that obtained previously with crude extracts of *C. reinhardtii* (Table 1) (Husic and Tolbert 1985) and close to the value observed for the related d-LDH from *Lactobacillus bulgaricus* (Le Bras and Garel 1991). Consequently, we annotate Cre07.g324550 as Cr-LDH1. Interestingly, recombinant His-tagged Cr-LDH1 also showed glyoxylate reductase activity, albeit with a lower catalytic efficiency, indicating a more wide-ranging role in metabolism than just the reduction of pyruvate (Table 1).

**Table 1:** Kinetic properties of His-tagged Cr-LDH1

Substrate	*K*_M_ (mM)	*k*_cat_ (s^–1^)	*k*_cat_/*K*_M_ (M^–1^ s^–1^)
Pyruvate*^a^*	1.46 ± 0.01	370 ± 2	(2.55 ± 0.07) × 10^5^
Glyoxylate*^a^*	62	60	1.0 × 10^3^
d-Lactate*^b^*	70	10	1.5 × 10^2^

Assays were conducted in reaction buffer (25 mM sodium phosphate, 250 mM NaCl, pH 7.5) containing 0.4–9 µg ml^–1^ enzyme in the presence of either *^a^*300 µM NADH or *^b^*300 µM NAD^+^ (Supplementary Fig. S10).

For pyruvate, the average values (± SD) are given for two independent protein preparations. A previous analysis of the reduction of pyruvate using *C. reinhardtii* crude extracts gave a *K*_M_ (pyruvate) of 1.1 mM (Husic and Tolbert 1985). No activity was detected using NADPH or NADP^+^ (data not shown).

**Fig. 1:**
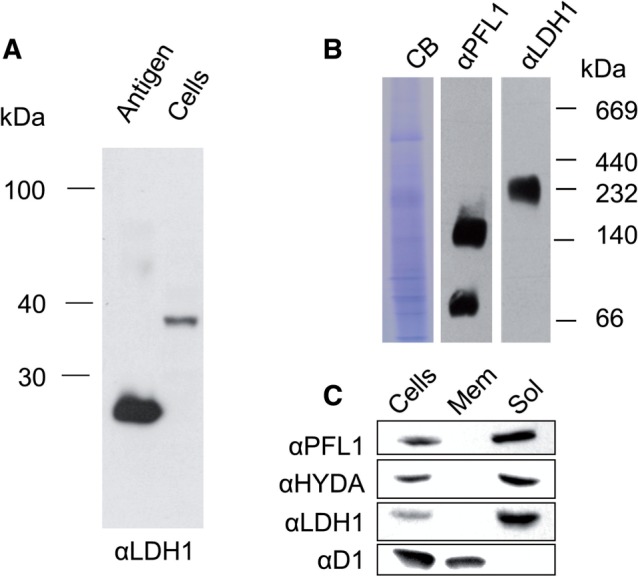
Immunochemical analysis of size and location of Cr-LDH1. (A) Immunoblot analysis of total *C. reinhardtii* cell extract (8.5 × 10^5^ cells loaded) using Cr-LDH1-specific antibodies (αLDH1) with approximately 10 ng of *E. coli*-expressed LDH1 antigen as control. (B) Analysis of Cr-LDH1 oligomeric state in whole-cell extracts separated by 1D BN-PAGE and probed with Cr-LDH1 antibodies (αLDH1). A PFL1 immunoblot (αPFL1) is provided for comparison, and loading is indicated by a Coomassie Brilliant Blue (CB)-stained gel. (C) Immunodetection of Cr-LDH1 in whole-cell extract (Cells) and in membrane (Mem) and soluble (Sol) fractions. Approximately 40 µg of protein was loaded per lane. HYDA, PFL1 and the D1 subunit of PSII were used as controls.

A polyclonal antibody raised against Cr-LDH1 detected a protein of approximately 40 kDa in cell extracts upon SDS–PAGE, in line with the predicted molecular mass (Fig. 1A). Blue native (BN) gels indicated that the Cr-LDH1 holoenzyme had an apparent mass of >200 kDa (Fig. 1B), suggesting the formation of a larger complex in vivo. In comparison, PFL1, which is known to form dimers (Becker et al. 1999), gave two bands consistent with the presence of monomeric (∼80 kDa) and dimeric forms (∼160 kDa) (Fig. 1B). As expected, Cr-LDH1, like PFL1 and HYDA1, was found to be a soluble protein, and was absent from the membrane fraction, which contained the D1 subunit of PSII (Fig. 1C).

### Structure of Cr-LDH1

**Fig. 2:**
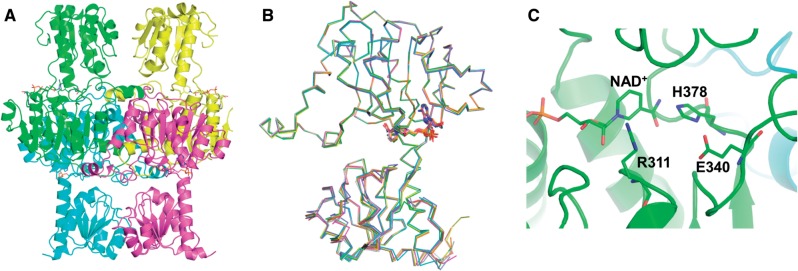
Crystal structure of His-tagged Cr-LDH1 (PDB:4ZGS). (A) Ribbon diagrams of the four chains proposed to form the tetramer. (B) Superposition of each of the four chains. NAD^+^ is shown in stick form. (C) View of NAD^+^ and the conserved histidine (H378), arginine (R311) and glutamate (E340) residues in the active site, shown in stick form. Numbering of residues is based on the decoded gene sequence (Supplementary Fig. S7). For the deposited structure, these residues are equivalent to H335, R268 and E297. Images were constructed using Pymol software.

His-tagged recombinant Cr-LDH1 was crystallized and its structure determined by X-ray crystallography (Supplementary Table S3). The co-ordinates and structure factors have been deposited in the PDB, under accession 4ZGS. There were eight copies of the monomer in the asymmetric unit, corresponding to two tetramers (Fig. 2A, B), as predicted by PISA (Krissinel and Henrick 2007). The overall structure of the individual chains was similar to previous structures of d-LDHs with, for example, a root mean square difference of 2.3 Å over 328 Cα atoms for chain A of Cr-LDH1 and the d-LDH enzyme from *L. bulgaricus* (PDB:1J49) (Supplementary Fig. S12). NAD^+^ molecules added during crystallization were observed in the active site of each monomer (Fig. 2C) close to the conserved His378, Arg311 and Glu340 residues noted previously (Supplementary Fig. S7).

### Generation of Cr-LDH1 knock-down mutants

**Fig. 3:**
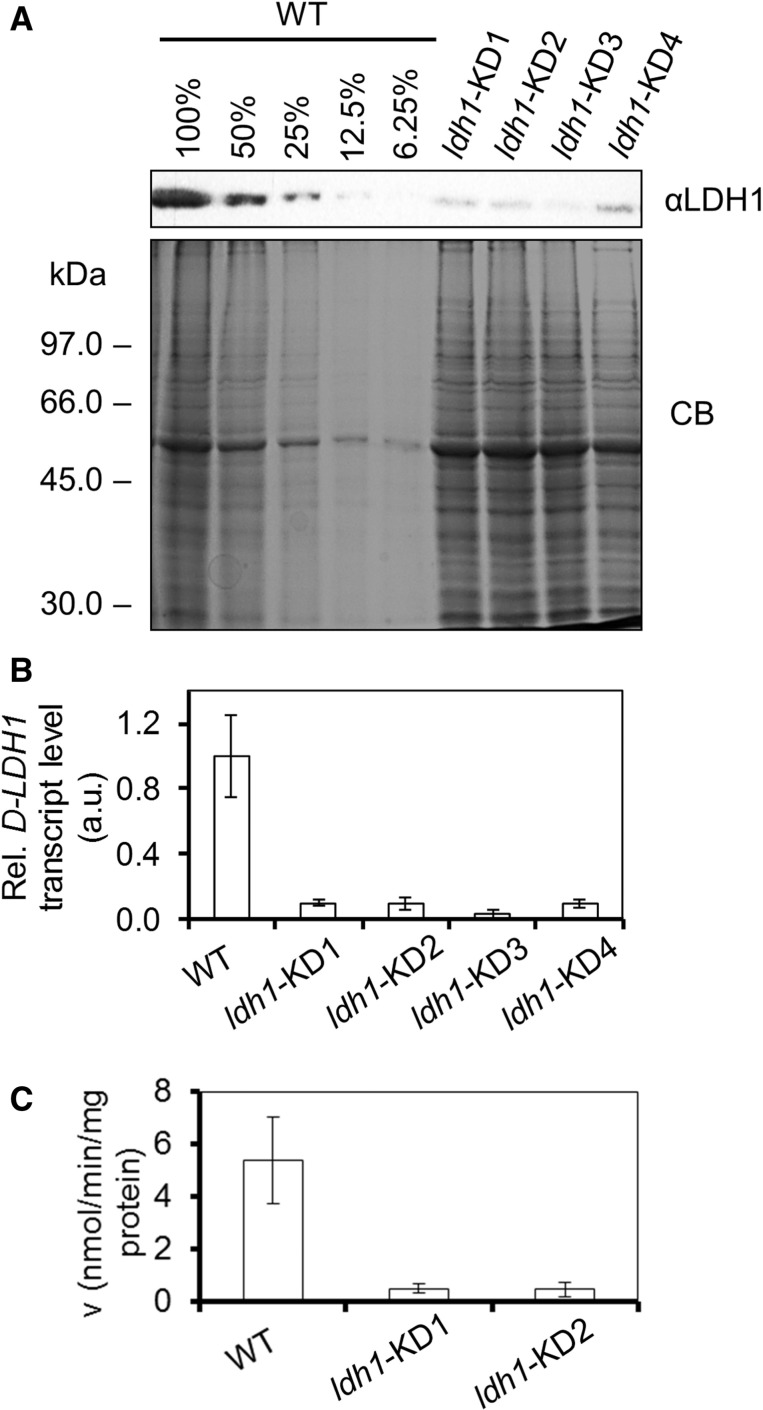
Comparison of CC-124 and *LDH1* knock-down cell lines grown in acetate-containing medium. (A) Immunoblot analysis of Cr-LDH1 levels (8.5 × 10^5^ cells loaded per lane). (B) Taqman® qRT–PCR analysis of *LDH1* mRNA levels. (C) LDH1 activity assays. Enzymatic activity (v) was calculated as nmol of NADH oxidized per minute per mg of total protein assayed. Error bars are given as ± SE of three biological replicates.

To test the contribution of Cr-LDH1 to d-lactate production *in vivo*, knock-down mutants were generated using an artificial microRNA (amiRNA) approach (Molnar et al. 2009). Four mutants were identified (named *ldh1*-KD1, 2, 3 and 4) with an approximately 80% reduction in the levels of both mRNA and protein (Fig. 3A, B). Activity assays revealed that the *ldh1*-KD1 and *ldh1*-KD2 knock-down strains, chosen for further study, had an approximately 80% decrease in total cellular pyruvate reductase activity, in line with the decrease in Cr-LDH1 levels (Fig. 3C), consistent with Cr-LDH1 being the dominant route for pyruvate reduction under the assay conditions used.

### Cr-LDH1 is present in the chloroplast

**Fig. 4:**
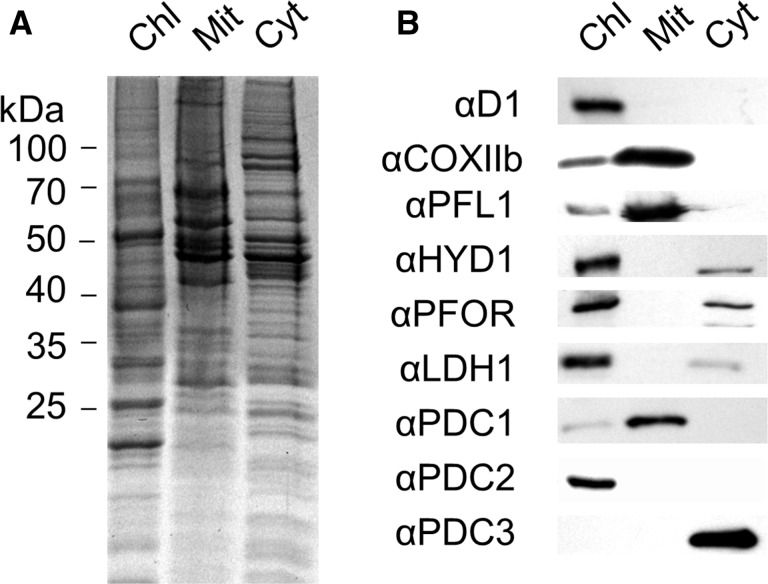
Determination of the subcellular distribution of pyruvate-degrading and fermentative enzymes in *C. reinhardtii*. Analysis of purified chloroplastic (Chl), mitochondrial (Mit) and cytoplasmic (Cyt) fractions, showing (A) a Coomassie Brilliant Blue (CB)-stained gel, (B) immunoblots. An 8 µg aliquot of protein was loaded per lane.

To determine the location of Cr-LDH1 in the cell, anoxia was induced in *C. reinhardtii* cultures and an immunoblot analysis was performed on isolated chloroplast, mitochondrial and cytoplasmic fractions (Fig. 4). Antibodies specific for the thylakoid D1 subunit of PSII and the mitochondrial cytochrome oxidase CoxIIB subunit indicated some residual low level mitochondrial contamination of the chloroplast fractions, but at levels seen in previous studies (e.g. Terashima et al. 2010).

Our immunochemical analysis clearly showed that Cr-LDH1 was located in the chloroplast, consistent with the presence of a predicted chloroplast transit peptide. Also detected immunochemically in the chloroplast fraction were the HYDA subunits and PFOR, all previously assigned as chloroplast enzymes (Terashima et al. 2010, van Lis et al. 2013) (Fig. 4B). Small amounts of HYDA1/2 and PFOR could also be detected in the cytoplasmic fraction, most probably because of contamination by stromal proteins.

Importantly, we were able to show that PDC3 was present in the cytoplasmic fraction (Fig. 4B), which explains why previous proteomic analyses of chloroplast and mitochondrial fractions had only detected this protein at trace levels (Atteia et al. 2009, Terashima et al. 2010).

PFL1 has previously been suggested to be co-localized to the mitochondrion and chloroplast (Atteia et al. 2006); here the protein was predominantly present in the mitochondrion (Fig. 4B), with a weaker signal detected in the chloroplast (Fig. 4B) when assessed on an equal protein basis. However, immunogold localization of PFL1 suggested that the chloroplast signal is likely to be genuine, supporting co-localization (Supplementary Fig. S13).

PDC1 and PDC2 were found exclusively in the mitochondrion and chloroplast, respectively (Fig. 4B), consistent with their participation in organelle-specific PDH complexes (Shtaida et al. 2014) (Supplementary Fig. S1).

### Cr-LDH1 expression during mixotrophic and heterotrophic growth

**Fig. 5:**
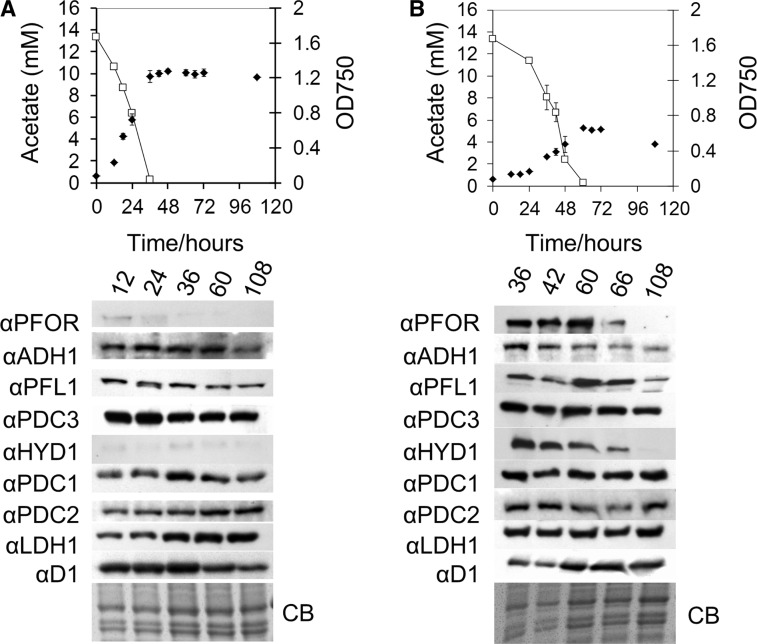
Analysis of fermentative enzyme expression during growth in acetate-containing medium. Cultures were grown in the light (A) or dark (B). Samples were taken from early log to late stationary phase at the indicated times. Top panels: line graphs comparing growth as estimated by OD_750_ (filled diamonds) against acetate concentration (open squares), error bars are provided ± SD of three biological replicates. Bottom panels: immunoblot analysis comparing abundance of fermentative enzyme expression over time; approximately 8 × 10^5^ cells were loaded per lane. Cultures were inoculated from mid-log phase TAP-grown cultures under continuous illumination. Protein loading is indicated by the Coomassie Brilliant Blue (CB)-stained gel.

Protein extracts were taken from cultures of wild-type (WT; CC-124) *C. reinhardtii* during growth in acetate-containing medium, either mixotrophically under continuous illumination, or heterotrophically in darkness, and analyzed by immunoblotting to look for changes in the expression of Cr-LDH1 and other fermentative enzymes as a function of growth phase (Fig. 5). The relative intensity of cross-reactions between light- and dark-grown cultures could be compared as samples were loaded on the same protein gel (Fig. 5). Relative to dark-grown cultures, the HYDA and PFOR proteins were poorly expressed in the light (Fig. 5A), but were induced during early growth stages in the dark (Fig. 5B), before declining at the onset of the stationary phase, which coincided with the depletion of acetate from the medium. In contrast, Cr-LDH1 as well as the other enzymes examined (ADH1, PFL1, PDC1, PDC2 and PDC3) was found to be present in both the light and the dark and at all stages of growth (Fig. 5).

### Expression of Cr-LDH1 during photoautotrophic growth

**Fig. 6:**
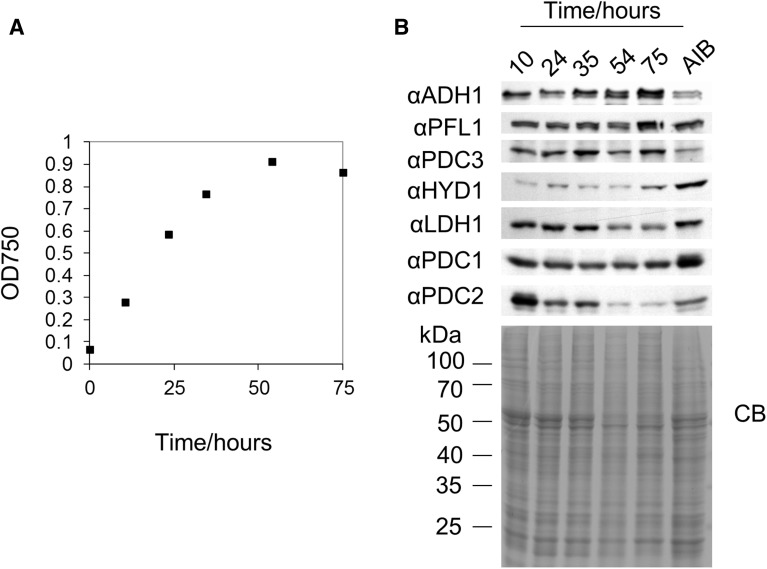
Analysis of fermentative enzyme expression during photoautotrophic growth. (A) Growth curve measuring OD_750_. (B) Immunoblot of fermentative enzyme expression during photoautotrophic growth compared with a sample of mid-log phase cells incubated in anaerobic induction buffer (AIB), in the dark, for 4 h with continuous argon purging. A total of 8 × 10^5^ cells were loaded per lane and loading was assessed by the Coomassie Brilliant Blue (CB)-stained gel.

To test whether Cr-LDH1 and other fermentative enzymes accumulated in cells in the presence of oxygen, WT (CC-124) cultures were grown in minimal medium with continuous aeration and samples were taken at 24 h intervals for immunoblot analysis (Fig. 6; Supplementary Fig. S14A). Under these growth conditions, the medium was largely saturated with oxygen as determined using a Clark electrode (Supplementary Fig. S14B). Enzyme expression was compared with that in cells grown in acetate medium that had been incubated in anaerobic induction buffer (AIB) in the dark for 4 h with continuous argon purging to induce anoxia.

As with the acetate-grown cultures (Fig. 5), most of the fermentative enzymes were constitutively present (Fig. 6B). Significant differences in enzyme expression during growth could only be observed for the putative chloroplast PDH complex protein PDC2, which showed a consistent decrease in levels from early log to stationary phase cultures (Fig. 6B; Supplementary Fig. S14A).

Interestingly, the [Fe–Fe]-hydrogenase was detected at low levels at all growth stages under photoautotrophic conditions (Fig. 6B; Supplementary Fig. S14A), although the enzyme is likely to be inactive owing to its oxygen sensitivity (Stripp et al. 2009).

### Cr-LDH1 expression under sulfur depletion in the light

**Fig. 7:**
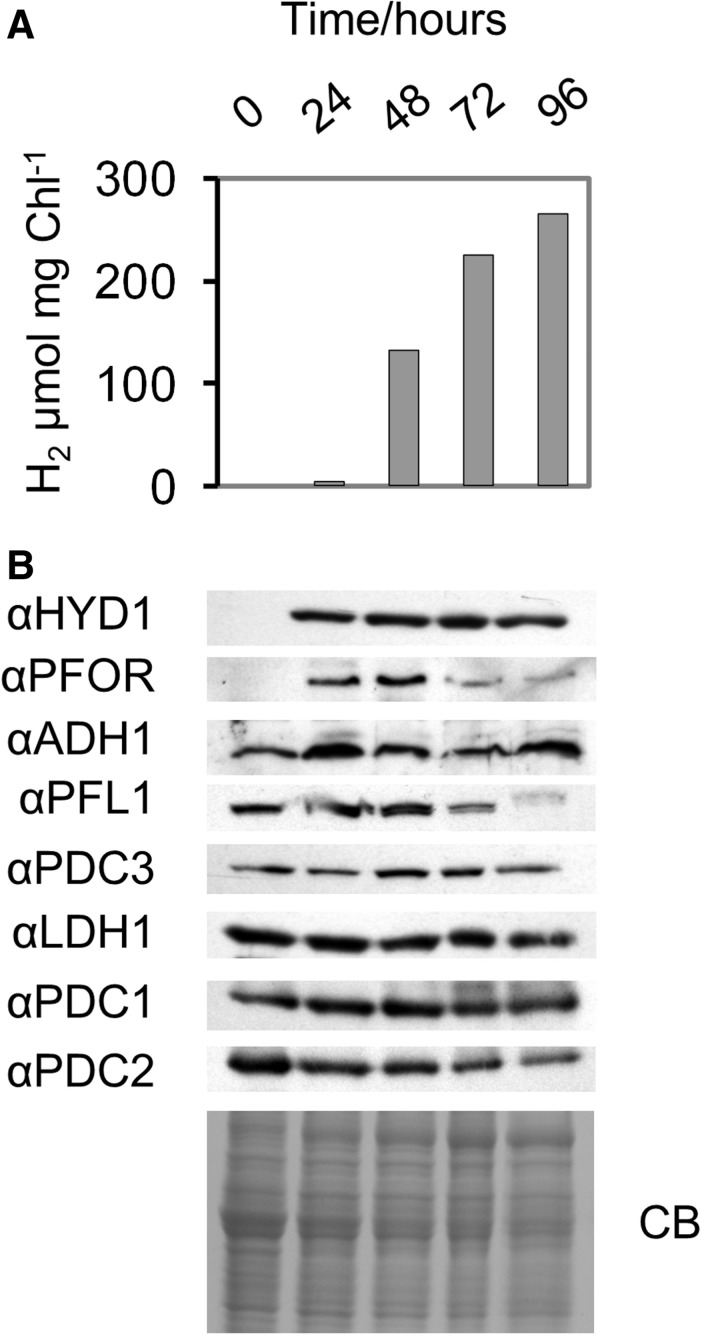
Analysis of fermentative enzyme expression during sulfur deprivation in the light. (A) H_2_ evolution measured by gas chromatography. (B) Immunoblot analysis with 8 × 10^5^ cells loaded per lane and protein loading assessed by Coomassie Brilliant Blue (CB). The zero time point was taken immediately prior to sulfur deprivation to avoid induction of hydrogenase.

A common method for the sustained production of hydrogen is to expose cells in sealed containers to light in an acetate-containing medium depleted in sulfur (Melis et al. 2000). Under these conditions, PSII activity becomes chronically photoinhibited so that the culture is driven sufficiently anaerobic in the presence of ongoing mitochondrial respiration to express the hydrogenase. Fig. 7A shows that, under the experimental conditions used, hydrogen production could be detected after 24 h of illumination, as could the accumulation of the HYDA subunits and PFOR (Fig. 7B), which are both induced under anoxic conditions at the transcriptional level (Mus et al. 2007). In contrast, changes in the protein levels of Cr-LDH1, as well as of ADH1, PFL1, PDC1, PDC2 and PDC3, were much less dramatic (Fig. 7B).

### Growth and metabolite production in knock-down mutants

As Cr-LDH1 was present in *C. reinhardtii* cultures under all growth conditions, we conducted growth assays on the knock-down mutants to assess a possible impact on cell fitness. All mutants grew photoautotrophically, mixotrophically and heterotrophically as well as the WT and showed no light sensitivity in growth (Supplementary Fig. S15).

**Fig. 8:**
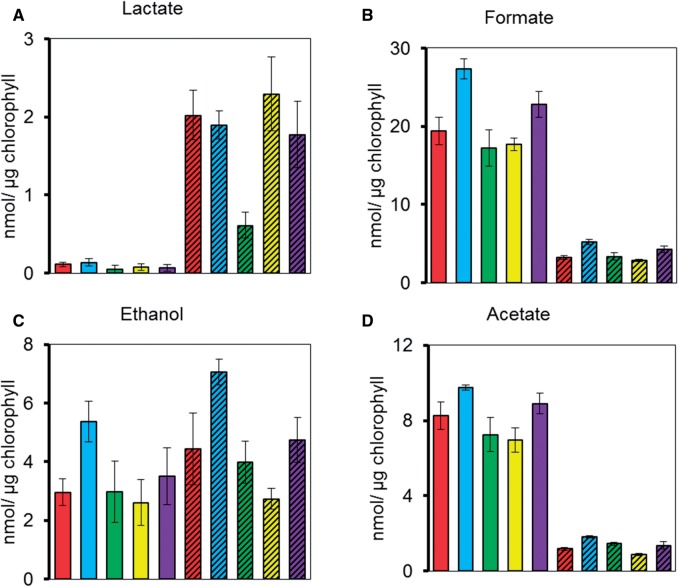
NMR analysis of excreted metabolites after 4 h of dark anaerobic incubation in high salt medium (HSM). Represented are CC-124 (red bars), *ldh1*-KD1 (blue bars), *ldh1*-KD2 (green bars), *ldh1*-KD3 (yellow bars) and *ldh1*-KD4 (purple bars). To inhibit PFL1, 10 mM sodium hypophosphite was added into the cultures (bars with diagonal lines). Error bars are given as ± SE of three biological replicates. Production of exclusively the d-lactate stereoisomer was confirmed using an enzyme assay.

To assess the impact of reduced Cr-LDH1 activity on the excretion of metabolites into the medium under anoxic conditions, cells of the *ldh1*-KD mutants were incubated in the dark in the presence of argon. Nuclear magnetic resonance (NMR) spectroscopy revealed that knock-down of Cr-LDH1 had no significant effect on the production of formate, ethanol and acetate after 4 h of dark anaerobic treatment (Fig. 8). Lactate is known to be excreted under anoxic conditions when formate production is blocked (Kreuzberg 1984, Philipps et al. 2011, Burgess et al. 2012, Catalanotti et al. 2012). Surprisingly, in the presence of sodium hypophosphite, a known inhibitor of PFL1, lactate was still produced in the knock-down mutants at WT levels (Fig. 8A), possibly due to the residual Cr-LDH1 activity in the mutants or the operation of other pathways. Immunoblotting confirmed that Cr-LDH1 levels were still knocked down under these experimental conditions (Supplementary Fig. S16) and enzyme assays confirmed the production of the d-stereoisomer (data not shown).

## Discussion

**Fig. 9:**
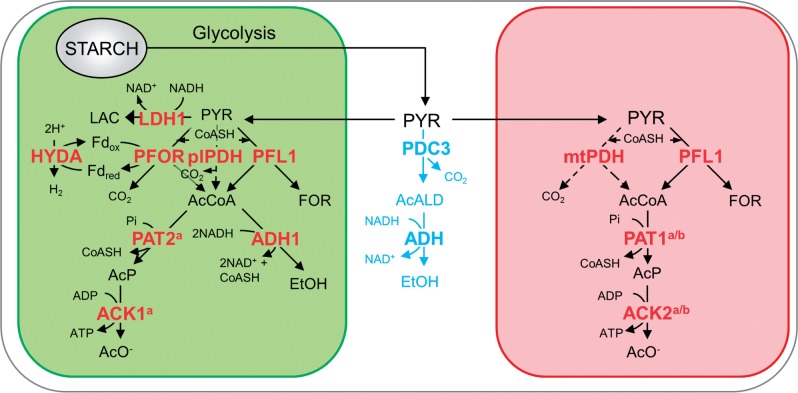
Proposed model of the subcellular localization of fermentative pathways in *C. reinhardtii*. Compartments within the cell are displayed: chloroplast (green), mitochondrion (red), cytoplasm (white). Highlighted are aerobic pathways (dashed line) and the putative cytoplasmic pyruvate degradation pathway (light blue). Abbreviations: Fd_ox_, ferredoxin (oxidized); Fd_red_, ferredoxin (reduced); AcALD, acetaldehyde; AcCoA, acetyl-CoA; AcP, acetyl-phosphate; CoASH, acetyl-CoA; EtOH, ethanol; FOR, formate; AcO^–^, acetate; LAC, lactate; PYR, pyruvate. The annotated genes are: ACK1, acetate kinase 1 (Cre09.g396700); ACK2, acetate kinase 2 (Cre17.g709850); ADH1, acetaldehyde/alcohol dehydrogenase; HYDA1, [Fe–Fe]-hydrogenase (Cre20.g758200); LDH1, d-lactate dehydrogenase (Cre07.g324550); PAT2, phosphate acetyltransferase 2 (Cre09.g396650); plPDH, plastid pyruvate dehydrogenase complex; mtPDH, mitochondrial pyruvate dehydrogenase complex; PDC3, pyruvate decarboxylase 3 (Cre03.g165700); PFL1, pyruvate formate lyase (Cre01.g044800); PFOR, pyruvate:ferredoxin oxidoreductase (Cre11.g473950). Where specific designations have not been given, there are multiple isoforms present in *C. reinhardtii* and the precise enzyme involved has not yet been verified. ^a^As localized by Terashima et al. (2010). ^b^As identified by Atteia et al. (2009). A number of additional pathways are thought to be active in production of intracellular metabolites but were not analyzed here and have been excluded for simplicity; see Subramanian et al. (2014) for details. Additionally the glycolytic pathway has been omitted; see Klein (1986) for details of partitioning between the chloroplast and cytoplasm.

Early work by Husic and Tolbert (1985) first identified the presence of a pyruvate reductase activity in *C. reinhardtii* chloroplasts. Here we have extended these studies to identify the gene product responsible, to provide detailed structural analysis of the enzyme and to characterize its expression under various growth regimes. In addition, our immunochemical data have revealed that PDC3, a likely pyruvate decarboxylase, is located in the cytoplasm. Based on these results, we propose a refined spatial model for pyruvate dissimilation in *C. reinhardtii* (Fig. 9).

Our immunochemical analysis supports the view that Cr-LDH1 as well as most of the other enzymes implicated in pyruvate metabolism (ADH1, PFL1, PDC1, PDC2 and PDC3) is expressed under photoautotrophic (oxic) conditions (Atteia et al. 2006, Whitney et al. 2011, Magneschi et al. 2012, Whitney et al. 2012). The clear exceptions are the highly oxygen-sensitive PFOR (van Lis et al. 2013) and HYDA1/2 enzymes (Stripp et al. 2009), which are strongly regulated at the transcriptional level (Mus et al. 2007). This makes sense from a physiological perspective, as fermentative metabolism needs to respond rapidly to fluctuations in the local environment whereas hydrogen production needs sustained anoxia, such as during the night-time. PFL1 is known to be post-translationally activated by the PFL-activating enzyme (Atteia et al. 2006); whether LDH1, PDC3 and ADH1 are regulated post-translationally is currently unknown.

One possible role for Cr-LDH1 during aerobic growth might be to act as a chloroplast lactate valve to dissipate reducing equivalents via the synthesis of lactate, akin to the malate valve of higher plant chloroplasts (Scheibe 2004). In this scenario, NADPH produced by the light reactions would be converted to NADH for use by Cr-LDH1 via a transhydrogenase such as ferredoxin-NADP^+^ reductase (Voordouw et al. 1983, Spano and Schiff 1987). d-Lactate could be transported to the mitochondrion where it would be re-oxidized to pyruvate by NAD^+^-independent d-LDH enzymes and glycolate dehydrogenase, with the resulting reducing equivalents consumed by the respiratory chain. Indeed, isolated *C. reinhardtii* mitochondria have been shown to take up d-lactate (Beezley et al. 1976), and significant intracellular quantities of lactate have been detected in *C. reinhardtii*, which decrease during high light treatment (see the supplementary data of Davis et al. 2013). These are coincident with up-regulation of a mitochondrial glycolate dehydrogenase (Davis et al. 2013), which has a catalytic efficiency for d-lactate similar to that for glycolate (Aboelmy and Peterhansel 2014). Analysis of the genome sequence has also revealed two potential FAD-dependent, NAD-independent d-lactate dehydrogenases (d-iLDH; EC 1.1.2.4) (Supplementary Figs. S8, S9) possessing the FAD-binding domain 4 and C-terminal oxidase domain characteristic of d-iLDHs (http://pfam.sanger.ac.uk) (Supplementary Figs. S8, S9) (Pallotta et al. 2004, Engqvist et al. 2009). A lactate valve would be expected to prevent damage to the photosynthetic apparatus, especially PSII, under conditions of high light stress (Vass 2012). However, the knock-down mutants constructed here, expressing 10–20% of WT levels of Cr-LDH1, behaved like the WT under high light (Supplementary Fig. S17), consistent with the presence of compensating pathways such as the malate shuttle (Scheibe 2004).

The presence of both lactate-producing (d-nLDH) and -consuming (d-iLDH and glycolate dehydrogenase) enzymes would also provide a mechanism for disposing of reducing equivalents under anoxia to maintain ATP production via glycolysis whilst leaving open the option of recapturing and re-metabolizing the excreted d-lactate once oxygen became available again. In this scenario, excretion of d-lactate into the medium might also be a mechanism for regulating internal pH levels. However, current data suggest that this is a pathway of last resort and only occurs when the production of formate and ethanol is blocked.

Likewise there may be a role for PDC3 under aerobic conditions. A cytosolic PDH complex bypass involving PDC has been described in yeast (Pronk et al. 1994), and a combined PDC/ADH pathway has been shown to regulate the pyruvate concentration in the roots of Arabidopsis under aerobic conditions, as a means of preventing excess respiration driving cells into an anoxic state (Zabalza et al. 2009). Current data indicate that only the chloroplast ADH1 enzyme is involved in ethanol production under anoxic conditions (Magneschi et al. 2012). Therefore, for PDC3 to be involved in ethanol production, a subpopulation of ADH1 would have to be present in the cytoplasm or acetaldehyde produced by PDC3 would be transported from the cytoplasm to ADH1 in the chloroplast. Alternatively, an additional ADH might be present in the cytoplasm that has so far escaped detection.

In accordance with the bioinformatic analysis, we have shown that PDC1 and PDC2, predicted to be components of larger PDH complexes, are located in the chloroplast and mitochondrion, respectively (Fig. 4). The role of the predicted chloroplast and mitochondrial PDH complexes in fermentation is still unclear, as they have not yet been purified. It is likely that pyruvate dissimilation via PDH complexes will predominate under aerobic conditions (Tadege et al. 1999), with fermentative pathways mainly activated during anoxia, as PDH complexes are inhibited by phosphorylation (mtPDH) (Patel and Korotchkina 2003, Dubini et al. 2009) and by the accumulation of NADH. However, a wider role for these complexes in pyruvate metabolism during anoxia cannot yet be ruled out.

Metabolite production in *ldh1*-KD lines was unaltered under anoxic conditions, which is consistent with this pathway acting as an overflow only at extremes of pH or when formate production is blocked (Kreuzberg 1984, Philipps et al. 2011, Burgess et al. 2012, Catalanotti et al. 2012). However, inhibition of formate production still resulted in significant excretion of d-lactate in the *ldh1*-KD lines analyzed here, which could be because the knock-downs were insufficient to limit flux to formate, perhaps via a compensatory increase in pyruvate levels, or because d-lactate can be produced via other routes such as from detoxification of methylglyoxal (MG), formed by de-phosphorylation of dihydroxyacetone phosphate (DHAP) (Cooper 1984). In bacteria, this reaction is catalyzed by methylglyoxyal synthase (MGS; EC 4.2.3.3) and (Saadat ad Harrison 1999), but in eukaryotic species where MGS is absent, MG can be formed by non-enzymatic decomposition of DHAP, with evidence for this coming from yeast (Martins et al. 2001), plants (Yadav et al. 2005, Chen and Thelen, 2010) and mammals (Thornalley 1996). MG is produced when DHAP accumulates and is then broken down by the activity of glyoxylase I (EC 4.4.1.5) and glyoxylase II (EC 3.1.2.6), candidate genes for which are encoded in the *C. reinhardtii* genome (Supplementary Fig. S18). Further work will be required to characterize this pathway in *C. reinhardtii*, but if it is responsible for continued accumulation of d-lactate it may complicate efforts at redirecting reductant towards H_2_ production.

## Materials and Methods

### Bioinformatic analysis

Transit peptide prediction was performed using online tools ChloroP (Emanuelsson et al. 1999) and TargetP (Emanuelsson et al. 2000). Protein sequence analysis was performed using the pfam database (Finn et al. 2014). *Chlamydomonas reinhardtii* expressed sequence tags (ESTs) were identified through the *Chlamydomonas* EST index (Asamizu et al. 2004) at the Kazusa DNA Research Institute (http://est.kazusa.or.jp/en/plant/chlamy/EST/index.html), BLAST analysis was performed using the Phytozome (Phytozome v10 http://www.phytozome.net/) (Goodstein et al. 2012) and NCBI databases (Madden, 2002).

### Strains and growth conditions

*Chlamydomonas reinhardtii* strains 137 c mt- (CC-124) and cw15 mt- (CC-406) were obtained from the Chlamydomonas Centre, Duke University, USA. Cells were grown in Tris acetate phosphate (TAP) medium (Gorman and Levine 1965) at 25°C and 60 µE m^–2^ s^–1^. For photoautotrophic growth, cells were cultured in high salt medium (HSM) (Sueoka 1960) under continuous illumination of 140 µE m^–2^ s^–1^ with continuous aeration. Cr-LDH1 knock-down mutants were generated by electroporating strain CC-124 with an Cr-LDH1 amiRNA vector as described in Burgess et al. (2012) and screening for reduced Cr-LDH1 expression by immunoblotting using Cr-LDH1 specific antibodies.

### Plasmid construction and RNA analysis

amiRNA vectors were created as described previously (Burgess et al. 2012). The oligonucleotides used for targeting Cr-LDH1 were 5′-ctagtGTGCTTGCCTATGACAACAAAtctcgctgatcggcaccatgggggtggtggtgatcagcgctaTTTGATGTCATAGGCAAGCACg-3′ (forward) and 5′-ctagcGTGCTTGCCTATGACATCAAAtagcgctgatcaccaccacccccatggtgccgatcagcgagaTTTGTTGTCATAGGCAAGCACa-3′ (reverse); in upper case is the amiRNA targeting sequence, in lower case are the amiRNA flanking and hairpin sequences. For Cr-LDH1 overexpression in *E. coli*, a codon-optimized Cr-LDH1 gene was synthesized (Biomatik). The open reading frame encoding the mature form of the protein (without the putative chloroplast transit peptide) was subcloned between the *Nco*I and *Xho*I sites of the pET28b expression vector (Novagen) to produce plasmid pET28b-LDH1. Analysis of *LDH1* mRNA levels by real-time quantitative reverse transcription–PCR (qRT–PCR) was performed as described previously (Burgess et al. 2012) using the primer sequences 5′-GCAGTGCTGTTCGTGAATGAC-3′ (forward), 5′-ACCCGCCTTGGCTAACTC-3′ (reverse) and 5′-ATGCGTCGGTGATCAA-3′ (Taqman® probe). Transcript levels were normalized to RPL10a mRNA using the primer sequences 5′-CCTGCTCCTATCAACAAGAACCT-3′ (forward), 5′-GAACTTGATGCTGCACTTGGT-3′ (reverse) and 5′-CCAGCACCATCTCCTC-3′ (Taqman® probe).

### Cr-LDH1 expression in *E. coli*

A C-terminal His-tagged derivative of Cr-LDH1 was expressed in *E. coli* Single Step (KRX) cells (Promega UK) transformed with pET28b-LDH1. A 100 ml LB liquid culture supplemented with 50 µg ml^–1^ kanamycin was grown at 37°C to an OD_600_ of approximately 0.5, then protein expression was induced by addition of l-rhamnose at 0.1% (w/v), and the culture was incubated at 30°C overnight. Overexpressed His-tagged Cr-LDH1 was purified from the bacterial cell lysate soluble fraction by immobilized metal ion affinity chromatography using Ni-Superflow Resin (Generon Ltd) at 4°C as described by Michoux et al. (2010). The affinity-bound sample was eluted with 400 mM imidazole and dialyzed against dialysis buffer (25 mM sodium phosphate, 250 mM NaCl, 1 mM EDTA, pH 7.5) to remove imidazole. The final solution of Cr-LDH1 was either used immediately in enzymatic activity assays or stored at 4°C. The oligomeric state of recombinant His-tagged Cr-LDH1 was assessed by gel filtration chromatography using a HiLoad 16/60 S-200 gel filtration column (GE Life Sciences) pre-equilibrated with running buffer (25 mM Tris, 250 mM NaCl, pH 7.5) and cooled via circulating water to approximately 8°C. The column was run at a flow rate of 1 ml min^–1^ and a single intense protein peak eluted at 65.8 ml. The molecular weight of the oligomer was estimated by comparison with a protein standard curve (Supplementary Fig. S10).

### Enzymatic activity assays

Kinetic traces were recorded on a UV-1601 UV-Visible spectrophotometer (Shimadzu UK Ltd) at 25°C using plastic cuvettes with a path length of 1 cm. Absorbance at 340 nm was recorded for the first 150 s of the reaction. An appropriate mass of purified recombinant Cr-LDH1 enzyme (0.4–9 µg) was combined with coenzyme at 300 µM and substrate at a specified concentration in reaction buffer (25 mM sodium phosphate, 250 mM NaCl, pH 7.5) in a total volume of 1 ml. Initial rates of the kinetics runs were recorded in either triplicate or duplicate and used to calculate the enzymatic parameters *K*_M_, *V*_max_, *k*_cat_ and *k*_cat_/*K*_M_. All analyses were performed using the data analysis and graphing software OriginPro 8.6 (OriginLab Corp.). The kinetic run plots were differentiated to give the rate d*A*_340_/d*t* at *t* = 0 for every substrate concentration, and these data were used to prepare Michaelis–Menten and Lineweaver–Burk plots. These in turn were fit with hyperbolic and linear functions, respectively, to yield the parameters *K*_M_ and *V*_max_. The concentration of Cr-LDH1 present in the kinetic assay, as determined by *DC* Protein Assay (Bio-Rad Laboratories Ltd) using bovine serum albumin (BSA) as a standard, was used to calculate *k*_cat_ and consequently *k*_cat_/*K*_M_. For endogenous Cr-LDH1 activity assays, TAP-grown cells were resuspended in 1/10th volume of buffer (25 mM sodium phosphate, 250 mM NaCl, pH 7.5) and lysed using a Vibra-Cell ultrasonic processor (Sonics & Materials Inc.). To remove cellular debris, the cell lysate was centrifuged (21,000 × *g*, 30 min, 4°C). The supernatant was collected and used immediately for enzyme assays. LDH activity was measured using 100 µl of cell extract, 300 µM NADH coenzyme and 10 mM sodium pyruvate substrate. A control assay was also carried out in the absence of sodium pyruvate, and the value obtained was used to subtract the background activity.

### Structure determination

His-tagged Cr-LDH1 was crystallized in a sitting drop arrangement by addition of 200 nl of protein/coenzyme solution at approximately 14.5 mg ml^–1^ protein and 300 µM NAD^+^ to 200 nl of reservoir solution [Wizard Classic crystallisation screen tube 8: 20% polyethylene glycol (PEG) 3350, 200 mM potassium nitrate] (Rigaku Europe). Several rhomboid-shaped crystals grew over the course of 10 d. An X-ray data set was recorded from one crystal on the I02 beamline at the Diamond Light Source synchrotron. Data were integrated and scaled with xia2 (Winter 2010) and programs of the CCP4 suite (Wynn at al. 2011). A total of 5% of reflections were set aside as the Free set for cross-validation. Data were processed to 2.46 Å, and the structure solved using molecular replacement with PHASER (McCoy et al. 2007) using d-LDH from *Lactobacillus helveticus* as a model (PDB code 2DLD). The structure was refined in PHENIX (Adams et al. 2010) with cycles of manual model building in COOT (Emsley et al. 2010). Validation was performed using the MolProbity server (Chen et al. 2010). The refined co-ordinates have been deposited at the Protein Data Bank with the accession code 4ZGS.

### Metabolite analysis

Excreted metabolites from *C. reinhardtii* were measured by NMR spectroscopy and biohydrogen was measured by gas chromatography (Burgess et al. 2012). A d/l-lactic acid assay kit (R-Biopharm AG) was used to determine the levels of excreted d- and l-lactate. Levels of acetate in the growth medium were determined by HPLC as described by Mus et al. (2007).

### Cell number calculation

For routine determination of *C. reinhardtii* cell number, a calibration curve was created relating the optical density at 750 nm, recorded on a UV-1601 UV-Visible spectrophotometer, to cell number, counted using a hemocytometer (Harris 1989). Cell number was subsequently calculated from OD_750_ measurements.

### Isolation of chloroplasts, mitochondria and cytoplasm

For chloroplast isolation, CC406 was grown in 12 h light/12 h dark cycles to a density of 0.6–1 × 10^7^ cells ml^–1^, and purged with argon for 4 h prior to harvesting, beginning 2 h into the light period of the third dark/light cycle. For isolation of mitochondria and chloroplasts, cells were harvested and broken according to Atteia et al. (2006) with the exception that a pressure of 30 psi was used to break cells that had been re-suspended to a density of 7 × 10^7^ cells ml^–1^, and a modified breaking buffer minus MgCl_2_ and MnCl_2_ was used. Intact mitochondria were subsequently isolated according to Eriksson et al. (1995), and chloroplasts were purified as described previously (Atteia et al. 2006) with the exception that intact chloroplasts were recovered from the interface of a two-step 40 : 60% Percoll gradient. To isolate the cytoplasmic fraction, cells re-suspended in breaking buffer (Atteia et al. 2006) were broken by passing through a 27 guage needle according to Mason et al. (2006) with the resulting lysate centrifuged to remove unbroken cells, chloroplasts and thylakoid membranes (650 × *g*, 30 min, 4°C). The supernatant was collected and spun down twice to remove any contaminating mitochondria (9,000 × *g*, 30 min, 4°C). The cytoplasmic proteins were collected from the resulting supernatant by mixing with 20% trichloroacetic acid at a 1 : 1 (v/v) ratio and incubating on ice for 20 min. The resulting precipitate was pelleted by centrifugation at maximum speed in a microfuge (13,000 r.p.m., 10 min, 4°C) and washed in –20°C acetone before centrifugation at maximum speed in a microfuge again (13,000 r.p.m., 5 min, room temperature). Acetone was removed and the pellet air dried before solubilizing in breaking buffer containing 10% (w/v) SDS.

### Antibody production

Antibodies were produced as previously described (Burgess et al. 2012). The ESTs used and the corresponding residues of the full-length protein chosen for expression are indicated: PDC3 (AV637523; residues 241–517), LDH1 (AV626597; residues 13–209), PDC1 (BG848457.1; residues 255–396), PDC2 (AV624259; residues 241–397), ADH1 (AV639995; residues197–368), PFOR (BP093588; residues 180–545). Anti-HYDA1 and anti-PFL1 have been created previously (Burgess et al. 2012).

### Gel electrophoresis and immunoblotting

SDS–PAGE, BN-PAGE and immunoblotting were performed according to Boehm et al. (2009).

## Supplementary data

Supplementary data are available at PCP online.

## Funding

This work was supported by the Engineering and Physical Sciences Research Council UK [EPSRC; research grant EP/F00270X/1]; the Government of Brunei Darussalam [a PhD scholarship to H.T.]; and the Biotechnology and Biological Sciences Research Council (BBSRC) [a David Phillips Fellowship (BB/F023308/1) to J.W.M.].

## Supplementary Material

Supplementary Data

## Abbreviations

ADH: alcohol dehydrogenase
amiRNA: artificial microRNA
BN: blue native
Cyt: cytochrome
EST: expressed sequence tag
HYD1: [Fe–Fe]-hydrogenase
LDH1: lactate dehydrogenase
NMR: nuclear magnetic resonance
PDC1: pyruvate dehydrogenase E1 α component 1
PDC2: pyruvate dehydrogenase E1 α component 2
PDC3: pyruvate decarboxylase
PFL1: pyruvate formate lyase
PFOR: pyruvate:ferredoxin oxidoreductase
PSI: photosystem one
PSII: photosystem two
TAP: Tris acetate phosphate
WT: wild type
