# Supplementary material for: Identification of the Elusive Pyruvate Reductase of Chlamydomonas reinhardtii Chloroplasts
Source: Plant Cell Physiol. 2015 Nov 15;57(1):82–94. doi: 10.1093/pcp/pcv167 (PMC4722173; doi:10.1093/pcp/pcv167)
Supplement: Supplementary Data [file supp_57_1_82__index.html]

Identification Of The Elusive Pyruvate Reductase Of Chlamydomonas reinhardtii Chloroplasts — Identification of the Elusive Pyruvate Reductase of Chlamydomonas reinhardtii Chloroplasts — Supplementary Data 

# Identification of the Elusive Pyruvate Reductase of *Chlamydomonas reinhardtii* Chloroplasts

## Supplementary Data

files

- Supplementary Data - zip file
