# Supplementary material for: Identification of the Elusive Pyruvate Reductase of Chlamydomonas reinhardtii Chloroplasts
Source: Plant Cell Physiol. 2015 Nov 15;57(1):82–94. doi: 10.1093/pcp/pcv167 (PMC4722173; doi:10.1093/pcp/pcv167)
Supplement: Supplementary Data [file supp_pcv167_suppl_data.zip › pcp-2015-e-00308-File024.pdf]

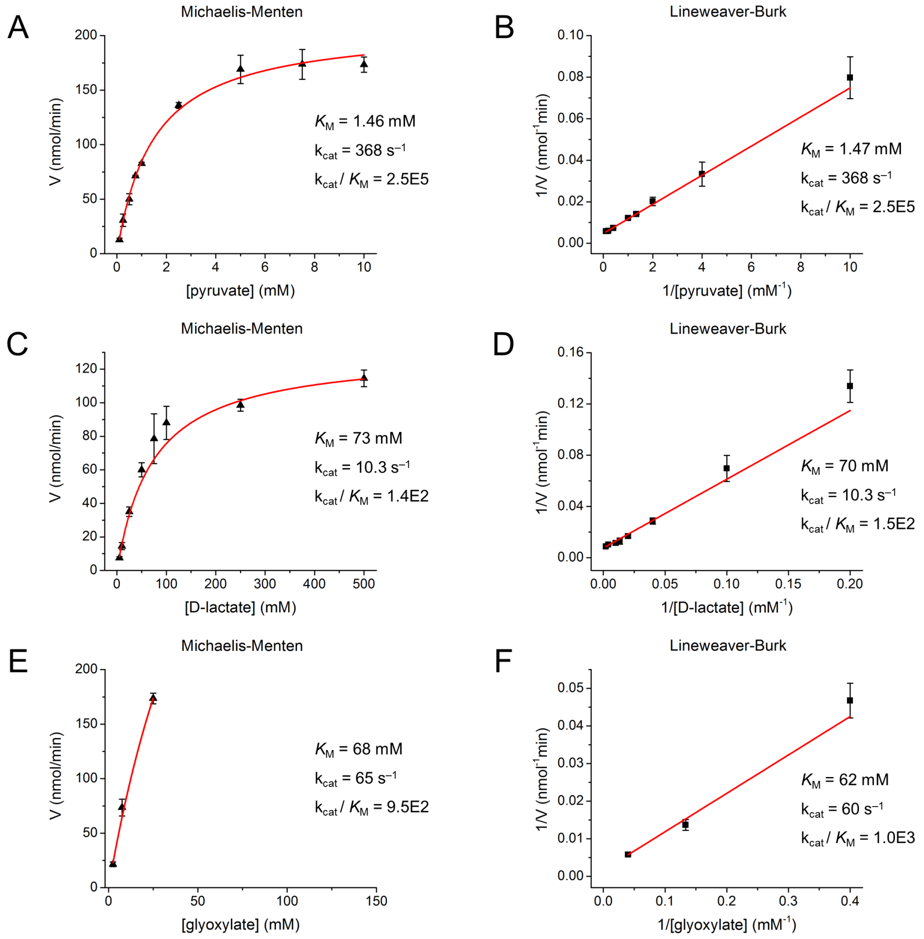

Figure S11: Kinetics data for recombinant *Cr*-LDH1. Three different potential substrates were tested: pyruvate (A and B), D-lactate (C and D) and glyoxylate (E and F). Initial rates were recorded over a range of substrate concentrations and the data plotted in Michaelis-Menten format and fit with a hyperbolic function (A, C and E) and Lineweaver-Burk format and fit with a linear function (B, D and F) to yield the kinetic parameters K_M and V_{max}.
