# Supplementary material for: Identification of the Elusive Pyruvate Reductase of Chlamydomonas reinhardtii Chloroplasts
Source: Plant Cell Physiol. 2015 Nov 15;57(1):82–94. doi: 10.1093/pcp/pcv167 (PMC4722173; doi:10.1093/pcp/pcv167)
Supplement: Supplementary Data [file supp_pcv167_suppl_data.zip › pcp-2015-e-00308-File025.pdf]

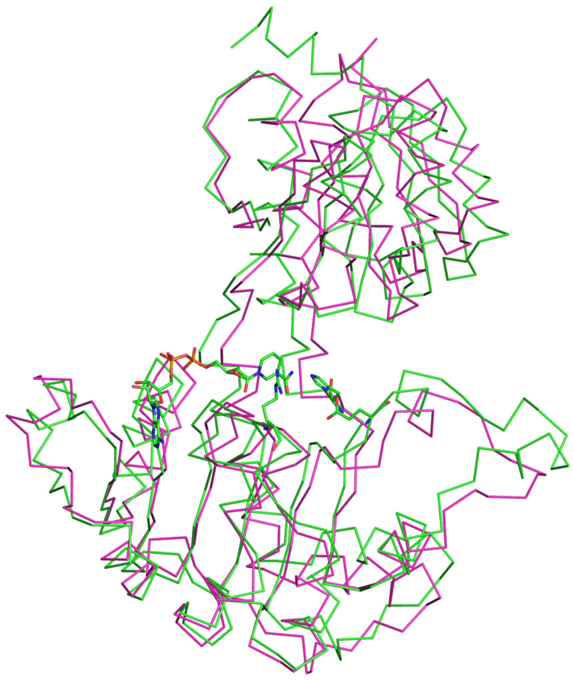

Figure S12: Figure showing alignment between Cr-DLDH1 (green) and DLDH from *L. bulgaricus*, PDB:1J49 (magenta), showing a 2.3Å RMSD over 328 Cα atoms. The active site NAD^+ and residues H378, R311 and Glu340 of Cr-LDH1 are shown in stick representation.
