# Supplementary material for: Identification of the Elusive Pyruvate Reductase of Chlamydomonas reinhardtii Chloroplasts
Source: Plant Cell Physiol. 2015 Nov 15;57(1):82–94. doi: 10.1093/pcp/pcv167 (PMC4722173; doi:10.1093/pcp/pcv167)
Supplement: Supplementary Data [file supp_pcv167_suppl_data.zip › pcp-2015-e-00308-File026.pdf]

A

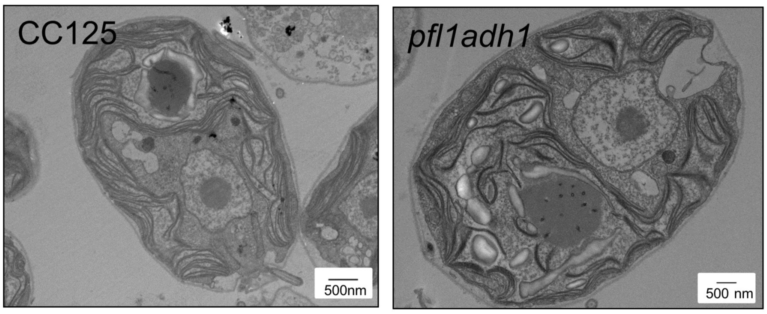

B

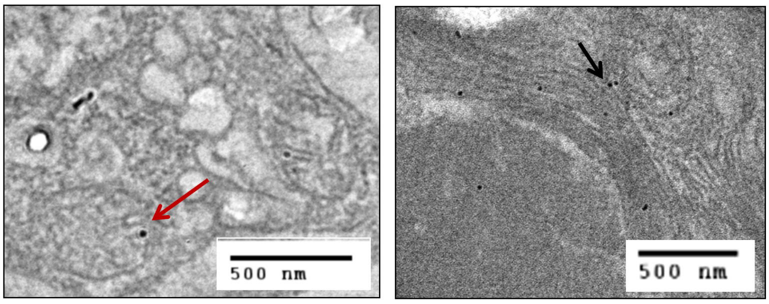

C

Organelle	Area (μm^2)	Density (Particles/ μm^2)	Particles per cell (%)
Mitochondrion	0.5 ± 0.1	4.5 ± 1.1	15
Chloroplast	13.1 ± 1.1	1.0 ± 3.6	85

Figure S13 Immunogold localisation (IGL) of PFL1. (A) Transmission electron microscopy (TEM) images of CC-125 and *pfl1adh1* after 4 h hypoxic induction. (B) IGL localisation of PFL1, example of signal located in mitochondria and chloroplast indicated by red and black arrow respectively. (C) Summary of statistics from IGL analysis, data was calculated by subtracting density of *pfl1adh1* signal from CC-125. 30 images were analysed for each line (2263 and 1801 spots counted for CC-125 and *pfl1adh1* respectively). Significant enrichment of PFL1 signal in CC-125 relative to *pfl1adh1* was observed in mitochondria (p-value = 0.0001604) and chloroplasts (p-value=0.003374).
