# Supplementary material for: Identification of the Elusive Pyruvate Reductase of Chlamydomonas reinhardtii Chloroplasts
Source: Plant Cell Physiol. 2015 Nov 15;57(1):82–94. doi: 10.1093/pcp/pcv167 (PMC4722173; doi:10.1093/pcp/pcv167)
Supplement: Supplementary Data [file supp_pcv167_suppl_data.zip › pcp-2015-e-00308-File027.pdf]

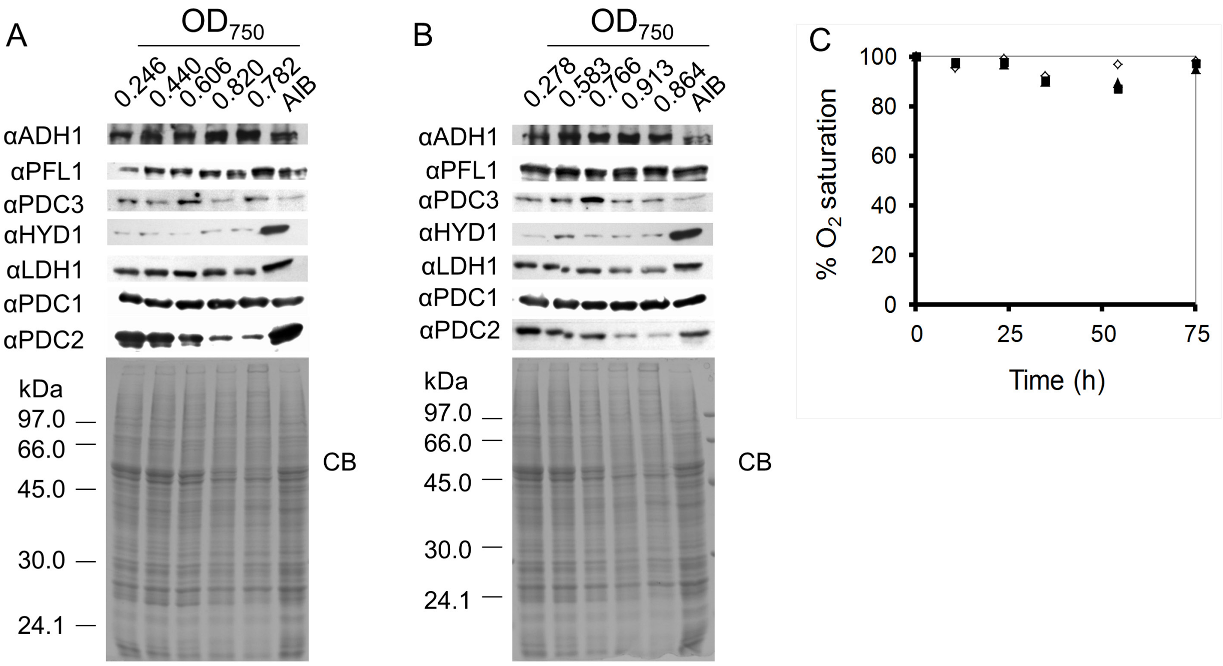

Figure S14: Replicate analysis of fermentative enzyme expression during photoautotrophic growth (for conditions see legend of Figure 5). Showing immunoblots on biological replicates 2 (A) and 3 (B), 8×10^5 cells loaded per lane, loading is indicated by CB stained gel. (C) % O_2 saturation measured by Clark electrode (Hansatech, UK) with two biological replicates plotted (1 in filled triangles, 2 in empty diamonds).
