# Supplementary material for: Identification of the Elusive Pyruvate Reductase of Chlamydomonas reinhardtii Chloroplasts
Source: Plant Cell Physiol. 2015 Nov 15;57(1):82–94. doi: 10.1093/pcp/pcv167 (PMC4722173; doi:10.1093/pcp/pcv167)
Supplement: Supplementary Data [file supp_pcv167_suppl_data.zip › pcp-2015-e-00308-File028.pdf]

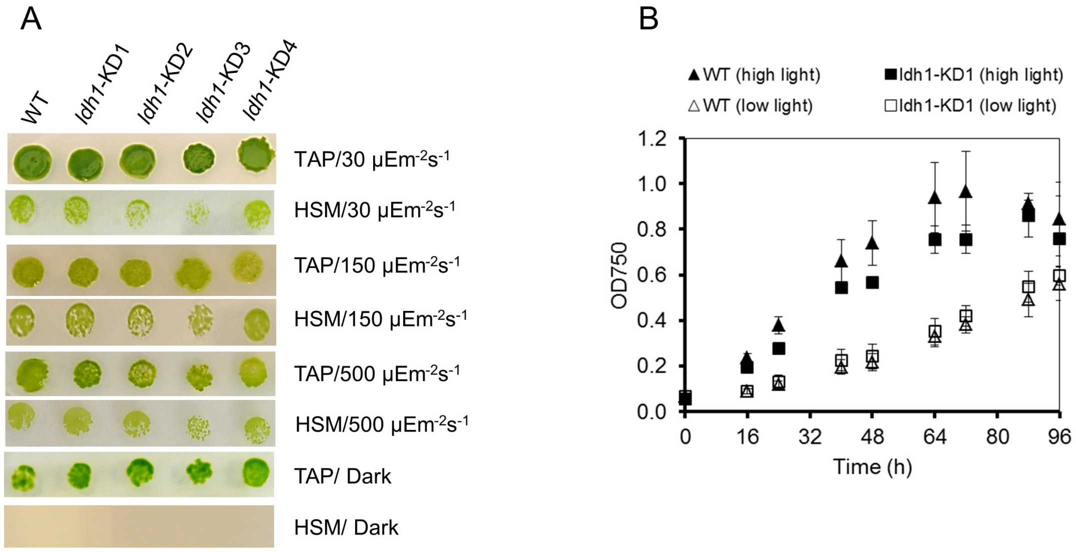

Figure S15: Growth assays for investigating *ldh1*-KD fitness. (A) Qualitative growth analysis: Equal number of cells were spotted either on a TAP or HSM plate, and exposed continuously to different light intensities. Photographs were taken after 1 to 2 weeks of growth. For each treatment, six biological replicates were carried out and a representative result is shown. (B) Quantitative growth analysis: Cells were grown in HSM that was bubbled with air and exposed continuously either to low (30 µE m^{-2} s^{-1}) or high light (150 µE m^{-2} s^{-1}). Similar results were obtained for all *ldh*-KD mutants and a representative result is shown. Error bars are given as ± SE of three independent biological replicates.
