# Supplementary material for: Identification of the Elusive Pyruvate Reductase of Chlamydomonas reinhardtii Chloroplasts
Source: Plant Cell Physiol. 2015 Nov 15;57(1):82–94. doi: 10.1093/pcp/pcv167 (PMC4722173; doi:10.1093/pcp/pcv167)
Supplement: Supplementary Data [file supp_pcv167_suppl_data.zip › pcp-2015-e-00308-File029.pdf]

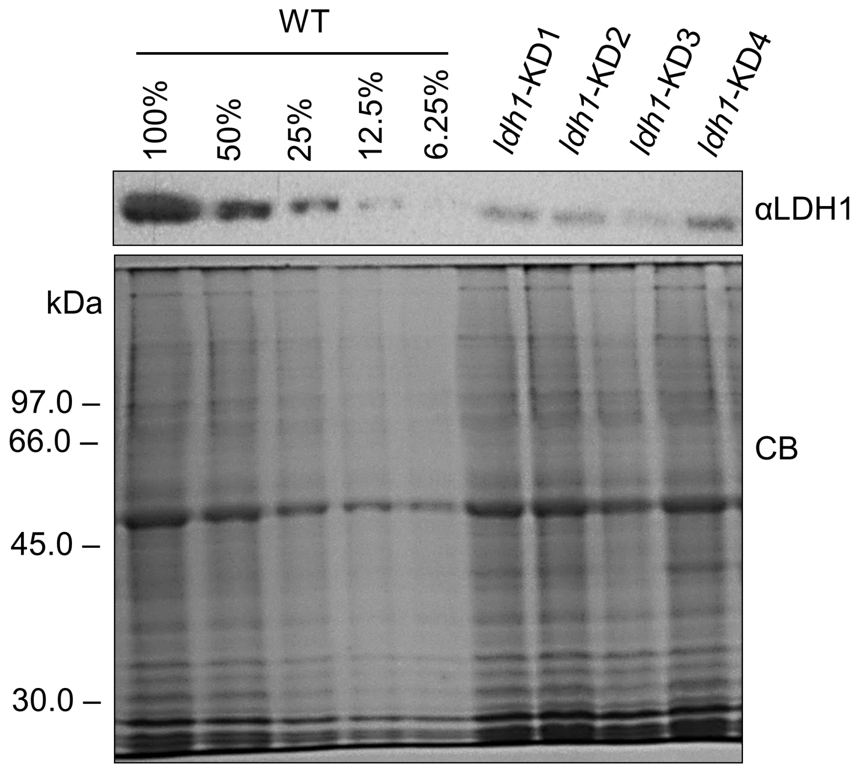

Figure S16: Immunoblot analysis of LDH1 levels after 4 h of dark anaerobic growth in HSM. Protein loading is indicated by CB stained gel, and 8.5×10^5 cells were loaded per lane.
