# Supplementary material for: Identification of the Elusive Pyruvate Reductase of Chlamydomonas reinhardtii Chloroplasts
Source: Plant Cell Physiol. 2015 Nov 15;57(1):82–94. doi: 10.1093/pcp/pcv167 (PMC4722173; doi:10.1093/pcp/pcv167)
Supplement: Supplementary Data [file supp_pcv167_suppl_data.zip › pcp-2015-e-00308-File030.pdf]

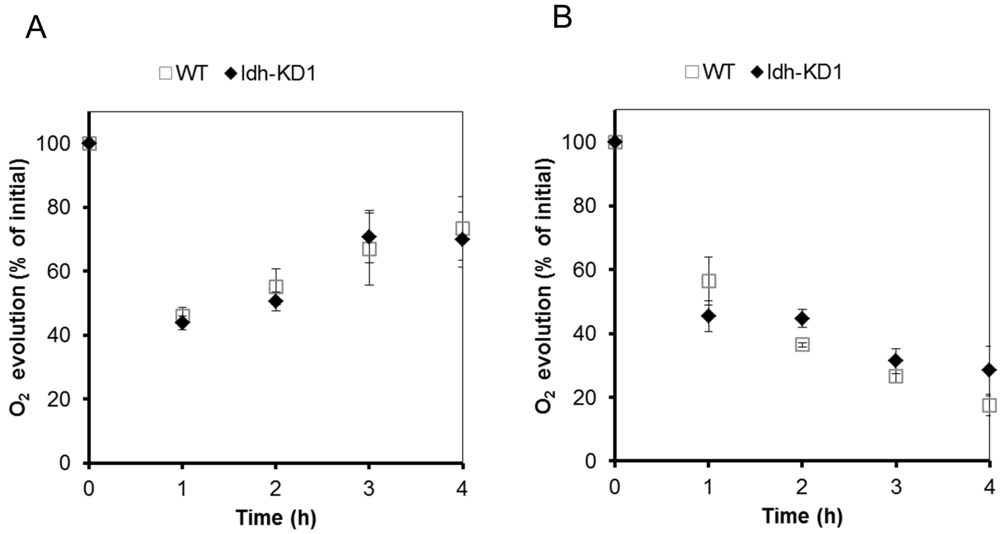

Figure S17: PSII repair assays. Light-saturated rate of oxygen evolution from HSM-grown cells exposed to 500 µE m^{-2} s^{-1} for a duration of 0 to 4 h. The cells were incubated in HSM either without (A) or with (B) 35 µg/ml chloramphenicol (to inhibit protein synthesis). Oxygen evolution was measured using a Clark O_2 electrode in the presence of 1 mM 2,5-dichloro-1,4-benzoquinone (DCBQ) and 1 mM potassium ferricyanide. Similar results were obtained for all *ldh*-KD mutants and a representative result is shown. Mean values (± SE) were based on three independent replicates and expressed as percentage of the initial values (40–60 µmole h^{-1} mg^{-1} chlorophyll).
