# Supplementary material for: Identification of the Elusive Pyruvate Reductase of Chlamydomonas reinhardtii Chloroplasts
Source: Plant Cell Physiol. 2015 Nov 15;57(1):82–94. doi: 10.1093/pcp/pcv167 (PMC4722173; doi:10.1093/pcp/pcv167)
Supplement: Supplementary Data [file supp_pcv167_suppl_data.zip › pcp-2015-e-00308-File031.pdf]

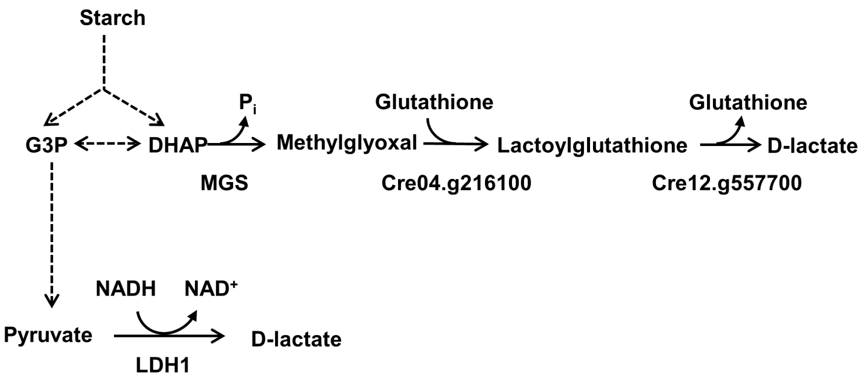

Figure S18: Proposed methylglyoxal pathway in *C. reinhardtii*. Methylglyoxal is formed by non-enzymatic elimination of phosphate (Pi) from glycolytic glyceraldehyde 3-phosphate (G3P) and dihydroxyacetone phosphate (DHAP) (Phillips & Thornalley, 1993). A clear homologue of methylglyoxal synthase (MGS, EC 4.2.3.3) is not encoded by the *C. reinhardtii* genome. Methylglyoxal is further converted to D-lactate by glyoxalase 1 (EC 4.4.1.5) and glyoxalase 2 (EC 3.1.2.6).
