# Supplementary material for: Identification of the Elusive Pyruvate Reductase of Chlamydomonas reinhardtii Chloroplasts
Source: Plant Cell Physiol. 2015 Nov 15;57(1):82–94. doi: 10.1093/pcp/pcv167 (PMC4722173; doi:10.1093/pcp/pcv167)
Supplement: Supplementary Data [file supp_pcv167_suppl_data.zip › pcp-2015-e-00308-File012.pdf]

Supplementary Methods

Strains: *C. reinhardtii137c* mt+ (CC-125) was obtained from the Chlamydomonas Centre, Duke University, USA and *pfl1adh1* double mutant (Catalanotti et al. 2012) was kindly provided by Arthur Grossman.

Bioinformatic analysis: For phylogenetic analysis putative transit sequences were removed prior to sequence alignment using MergeAlign (Collingridge and Kelly, 2012), Neighbour-joining trees (with midpoint rooting) were generated using EPoS software (Griebel et al. 2008). The numbers at the nodes are bootstrap values (1000 replicates). Protein alignments were performed using ClustalW (Larkin et al. 2007) and viewed using the GeneDoc software (Nicholas et al. 1997).

Analysis of dissolved O_2 concentration: This was performed according to Boehm et al. (2009).

Immunogold localisation (IGL) and Transmission Electron Microscopy (TEM). CC-125 and *pfl1adh1* were grown in TAP medium as described in methods. Cells were pelleted and incubated in the dark for 4h to induce hypoxic conditions. Samples were fixed and imaged at the Cambridge Advanced Imaging Centre according to (Genkov et al. 2010), with the exception that the anti-PFL1 antibody was used at a 1:10 dilution. ImageJ was used for visual analysis. Corrected Particle density (PD) for each strain was calculated $PD_{organelle} = PD_{organelle\_raw} - PD_{background}$. For each organelle the final particle density was calculated $PD_{final} = PD_{CC125} - PD_{pfl1adh1}$. The correct number of particles within the cell was then calculated by $PD_{final} *Area_{organelle}$. The percentage particles within the cell was calculated from the mean corrected particle numbers for each organelle. P-values for enrichment were determined by Wilcox test using R,
