# Supplementary material for: Identification of the Elusive Pyruvate Reductase of Chlamydomonas reinhardtii Chloroplasts
Source: Plant Cell Physiol. 2015 Nov 15;57(1):82–94. doi: 10.1093/pcp/pcv167 (PMC4722173; doi:10.1093/pcp/pcv167)
Supplement: Supplementary Data [file supp_pcv167_suppl_data.zip › pcp-2015-e-00308-File013.pdf]

Supplementary References

Collingridge, P. and Kelly, S. (2012) MergeAlign: improving multiple sequence alignment performance by dynamic reconstruction of consensus multiple sequence alignments. *BMC Bioinformatics* 13: 117.

Dereeper, A., Guignon, V., Blanc, G., Audic, S., Buffet, S., Chevenet, F., et al. (2008) Phylogeny.fr: robust phylogenetic analysis for the non-specialist. *Nucl. Acids Res.* 36: W465–W469.

Fries, M., Chauhan, H.J., Domingo, G.J., Jung, H.-I. and Perham, R.N. (2003) Site-directed mutagenesis of a loop at the active site of E1 (α2β2) of the pyruvate dehydrogenase complex. *Eur. J. Biochem.* 270: 861–870.

Genkov T, Meyer M, Griffiths H, Spreitzer RJ: (2010) Functional Hybrid Rubisco Enzymes with Plant Small Subunits and Algal Large Subunits: Engineered rbcS cDNA for expression in Chlamydomonas. *J. Biol. Chem.* 285:19833–19841.

Griebel, T., Brinkmeyer, M. and Bocker, S. (2008) EPoS: a modular software framework for phylogenetic analysis. *Bioinformatics* 24: 2399–2400.

Guindon, S., Lethiec, F., Duroux, P., Gascuel, O. (2005) PHYML Online - a web server for fast maximum likelihood-based phylogenetic inference. *Nucl. Acids Res.* 33: W557–W559.

Hawkins, C.F., Borges, A. and Perham, R.N. (1989) A common structural motif in thiamin pyrophosphate-binding enzymes. *FEBS Lett.* 255: 77–82.

Larkin, M.A., Blackshields, G., Brown, N.P., Chenna, R., McGettigan, P.A., McWilliam, H., et al. (2007) Clustal W and Clustal X version 2.0. *Bioinformatics* 23: 2947–2948.

Nicholas, K.B., Nicholas, H.B.Jr. and Deerfield, D.W.II. (1997) GeneDoc: analysis and visualization of genetic variation. *EMBNEW NEWS* 4: 14.

Phillips, S.A. and Thornalley, P.J. (1993) The formation of methylglyoxal from triose phosphates. *Eur. J. Biochem.* 212: 101–105.

Schenk, G., Leeper, F.J., England, R., Nixon, P.F. and Duggleby, R.G. (1997) The role of His113 and His114 in pyruvate decarboxylase from *Zymomonas mobilis*. *Eur. J. Biochem.* 248: 63–71.

Schneider, C.A., Rasband, W.S., Eliceiri, K.W: NIH Image to ImageJ: (2012) 25 years of image analysis. *Nat Meth,* **9**:671–675.

Siegert, P., McLeish, M.J., Baumann, M., Iding, H., Kneen, M.M., Kenyon, G.L., et al. (2005) Exchanging the substrate specificities of pyruvate decarboxylase from *Zymomonas mobilis* and benzoylformate decarboxylase from *Pseudomonas putida*. *Protein Eng. Des. Sel.* 18: 345–357.
