# Supplementary material for: Identification of the Elusive Pyruvate Reductase of Chlamydomonas reinhardtii Chloroplasts
Source: Plant Cell Physiol. 2015 Nov 15;57(1):82–94. doi: 10.1093/pcp/pcv167 (PMC4722173; doi:10.1093/pcp/pcv167)
Supplement: Supplementary Data [file supp_pcv167_suppl_data.zip › pcp-2015-e-00308-File014.pdf]

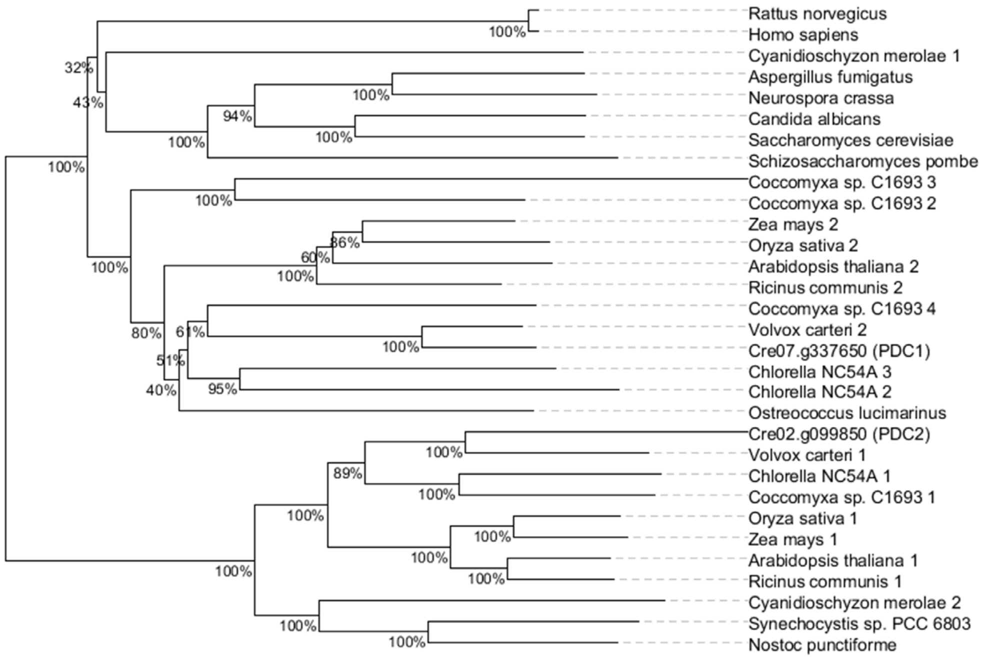

Figure S1: Phylogenetic analysis comparing *C. reinhardtii* PDC1 and PDC2 pyruvate dehydrogenase E1 alpha components with plant, fungal and mammal homologues. Branch support values calculated by aLRT test are represented in red (Guindon et al. 2005). Sequences of the putative *C. reinhardtii* PDH E1 α components (EC 1.2.4.1) PDC1 (Cre07.g337650) and PDC2 (Cre02.g099850) are compared to those of homologous enzymes from *Aspergillus fumigatus* (XP_750445.1), *Arabidopsis thaliana*[1] (NP_171617.1), *Arabidopsis thaliana*[2] (AAD39331.1), *Candida albicans* (EEQ44823.1), *Chlorella* NC54A[1] (32109[a]), *Chlorella* NC54A[2] (141042[a]), *Chlorella* NC54A[3] (56254[a]), *Coccomyxa* sp. C-1693[1] (33689[a]), *Coccomyxa* sp. C-1693[2] (29303[a]), *Coccomyxa* sp. C-1693[3] (73729[a]), *Coccomyxa* sp. C-1693[4] (25215[a]), *Cyanidioschyzon merolae*[1] (CMT256C[b]), *Cyanidioschyzon merolae*[2] (CMV153C[b]), *Homo sapiens* (NP_000275.1), *Neurospora crassa* (XP_957122.1), *Nostoc punctiforme* (WP_012411829.1), *Ostreococcus lucimarinus* (XP_001415646.1), *Oryza sativa subsp. indica*[1] (CAH65949.1), *Oryza sativa subsp. indica*[2] (EAZ00344.1), *Rattus norvegicus* (NP_001004072.2), *Ricinus communis*[1] (XP_002515074.1), *Ricinus communis*[2] (XP_002520198.1), *Saccharomyces cerevisiae* (EDN63155.1), *Schizosaccharomyces pombe* (NP_594892.1), *Synechocystis* PCC6803 (WP_010873216.1), *Volvox carteri f. nagariensis*[1] (XP_002950542.1), *Volvox carteri f. nagariensis*[2] (XP_002957475.1), *Zea mays*[1] (NP_001140759.1) *and Zea mays*[2] (NP_001150259.1). *C. reinhardtii* protein accessions are given according to the Phytozome database, all others refer to the NCBI database (http://www.ncbi.nlm.nih.gov/protein) unless [a]given as the JGI protein ID (http://www.jgi.doe.gov/), or [b]*Cyanidioschyzon merolae* genome project accession (http://merolae.biol.s.u-tokyo.ac.jp/).
