# Supplementary material for: Identification of the Elusive Pyruvate Reductase of Chlamydomonas reinhardtii Chloroplasts
Source: Plant Cell Physiol. 2015 Nov 15;57(1):82–94. doi: 10.1093/pcp/pcv167 (PMC4722173; doi:10.1093/pcp/pcv167)
Supplement: Supplementary Data [file supp_pcv167_suppl_data.zip › pcp-2015-e-00308-File015.pdf]

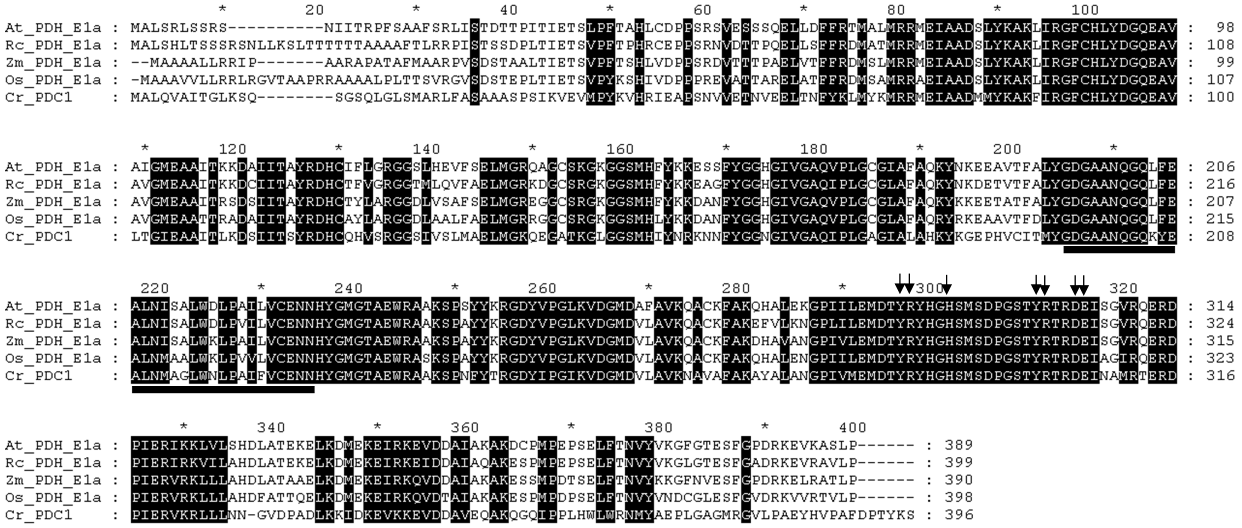

Figure S2: Protein sequence alignment comparing *Chlamydomonas reinhardtii* PDC1 (Phytozome ID: Cre07.g337650) with putative plant mitochondrial pyruvate dehydrogenase E1 alpha components. *Arabidopsis thaliana* (AAD39331.1), *Oryza sativa subsp. indica* (EAZ00344.1), *Ricinus communis* (XP_002520198.1), *Zea mays* (NP_001150259.1). Active site residues are indicated by black arrows (Fries et al. 2003) and the TPP binding motif GDG-X24-27-NN is underlined (Hawkins et al. 1989). Protein accessions given according to NCBI database (http://www.ncbi.nlm.nih.gov/protein).
