# Supplementary material for: Identification of the Elusive Pyruvate Reductase of Chlamydomonas reinhardtii Chloroplasts
Source: Plant Cell Physiol. 2015 Nov 15;57(1):82–94. doi: 10.1093/pcp/pcv167 (PMC4722173; doi:10.1093/pcp/pcv167)
Supplement: Supplementary Data [file supp_pcv167_suppl_data.zip › pcp-2015-e-00308-File017.pdf]

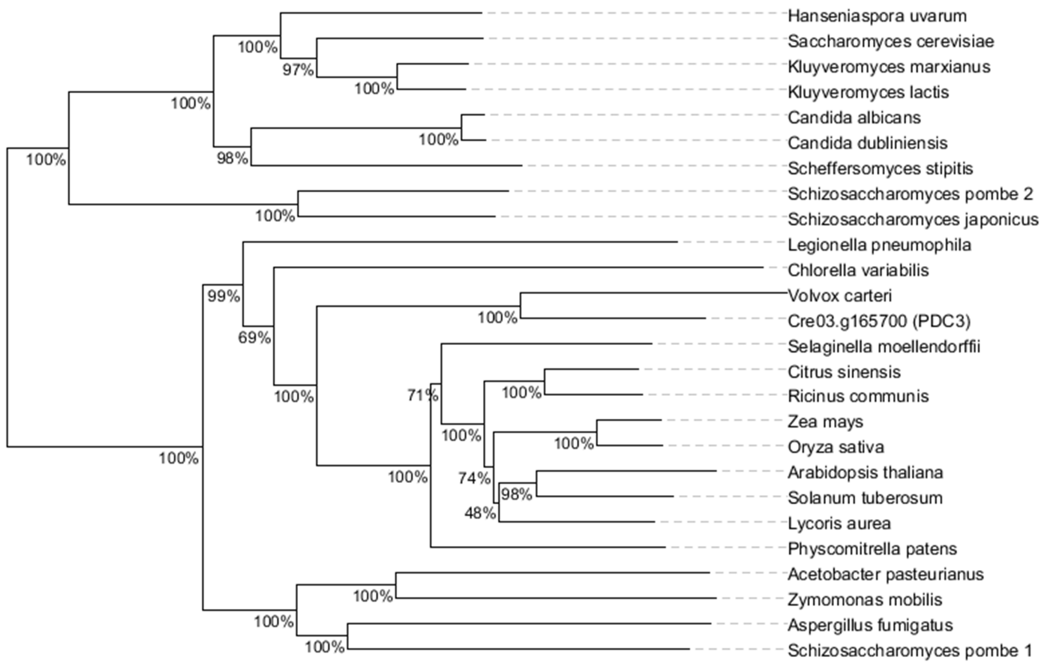

Figure S4: Phylogenetic analysis aligning putative *C. reinhardtii* PDC3 (XP_001703530.1) with pyruvate decarboxylase sequences (E.C.4.1.1.1): *Acetobacter pasteurianus* (AAM21208.1), *Arabidopsis thaliana* (NP_200307), *Aspergillus fumigatus* (XP_753176), *Candida albicans* SC5314 (XP_715589.1), *Candida dubliniensis* CD36 (XP_002420248.1), *Chlorella variabilis* (EFN55335.1), *Citrus sinensis* (AAZ05069.1), , *Hanseniaspora uvarum* (AAA85103.1), *Kluyveromyces lactis* NRRL Y–1140 (XP_454684.1), *Kluyveromyces marxianus* (AAA35267.1), *Legionella pneumophila* (YP_095188.1), *Lycoris aurea* (ABJ99597.1), *Oryza sativa subsp. japonica* (NP_001042088.1), *Physcomitrella patens subsp. patens* (XP_001763233.1), *Pichia stipitis* CBS 6054 (EAZ63546.2), *Ricinus communis* (XP_002522545.1), *Saccharomyces cerevisiae* (CAA39398.1), *Schizosaccharomyces japonicus* yFS275 (XP_002174876.1), *Selaginella moellendorffii* (XP_002986100.1), *Solanum tuberosum* (BAC23043.1), *Schizosaccharomyces pombe*[1] (NP_595027), *Schizosaccharomyces pombe*[2] 972h– (XP_001713041.1), *Volvox carteri f. Nagariensis* (XP_002951658.1), *Zea mays* (NP_001105645) and *Zymomonas mobilis* (AAA27685.1). Protein accessions given according to NCBI database (http://www.ncbi.nlm.nih.gov/protein).
