# Supplementary material for: Identification of the Elusive Pyruvate Reductase of Chlamydomonas reinhardtii Chloroplasts
Source: Plant Cell Physiol. 2015 Nov 15;57(1):82–94. doi: 10.1093/pcp/pcv167 (PMC4722173; doi:10.1093/pcp/pcv167)
Supplement: Supplementary Data [file supp_pcv167_suppl_data.zip › pcp-2015-e-00308-File018.pdf]

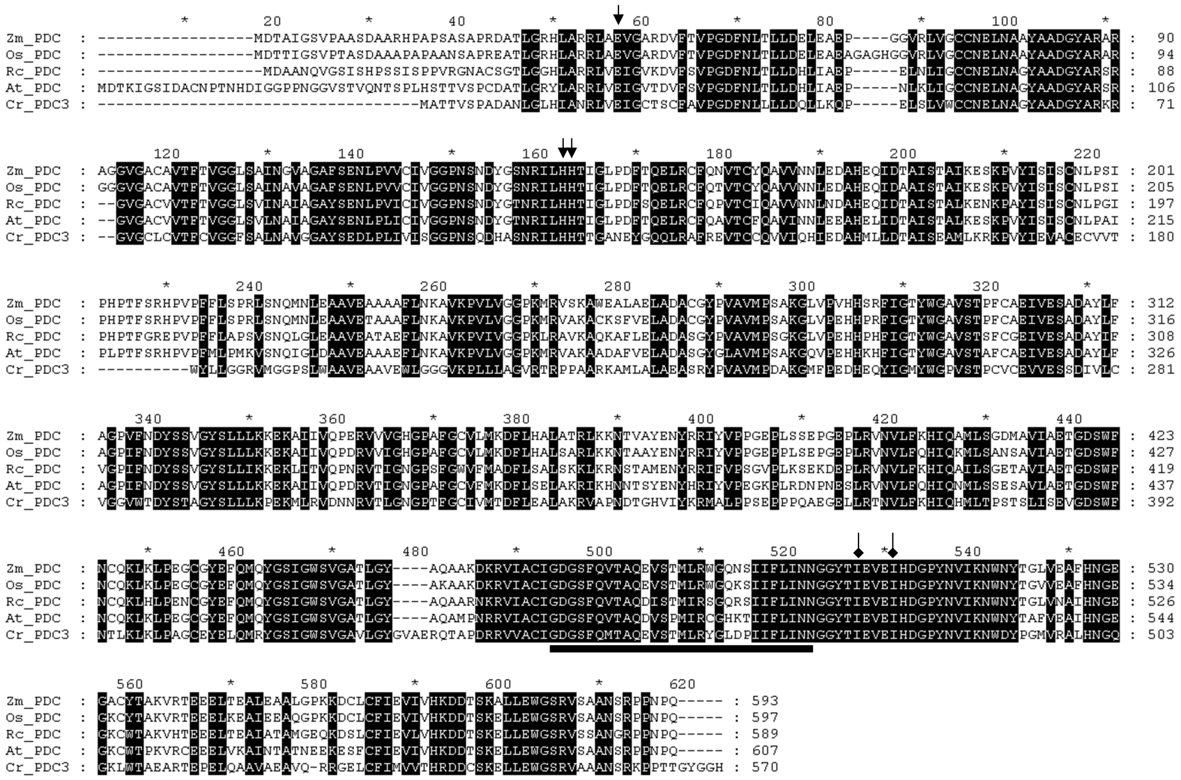

Figure S5: Protein sequence alignment comparing *C. reinhardtii* PDC3 (XP_001703530.1), with plant pyruvate decarboxylase sequences (EC 4.1.1.1). *Arabidopsis thaliana* (NP_200307), *Zea mays* (NP_001105645), *Oryza sativa subsp. japonica* (NP_001042088.1), *Ricinus communis* (XP_002522545.1). Active site histidine residues are indicated by black arrows (Schenk et al. 1997), TPP substrate recognition residues are indicated by black sticks (Siegert et al. 2005) and the TPP binding motif GDG–X24–27–NN is underlined (Hawkins et al. 1989). Protein accessions given according to NCBI database (http://www.ncbi.nlm.nih.gov/protein).
