# Supplementary material for: Identification of the Elusive Pyruvate Reductase of Chlamydomonas reinhardtii Chloroplasts
Source: Plant Cell Physiol. 2015 Nov 15;57(1):82–94. doi: 10.1093/pcp/pcv167 (PMC4722173; doi:10.1093/pcp/pcv167)
Supplement: Supplementary Data [file supp_pcv167_suppl_data.zip › pcp-2015-e-00308-File019.pdf]

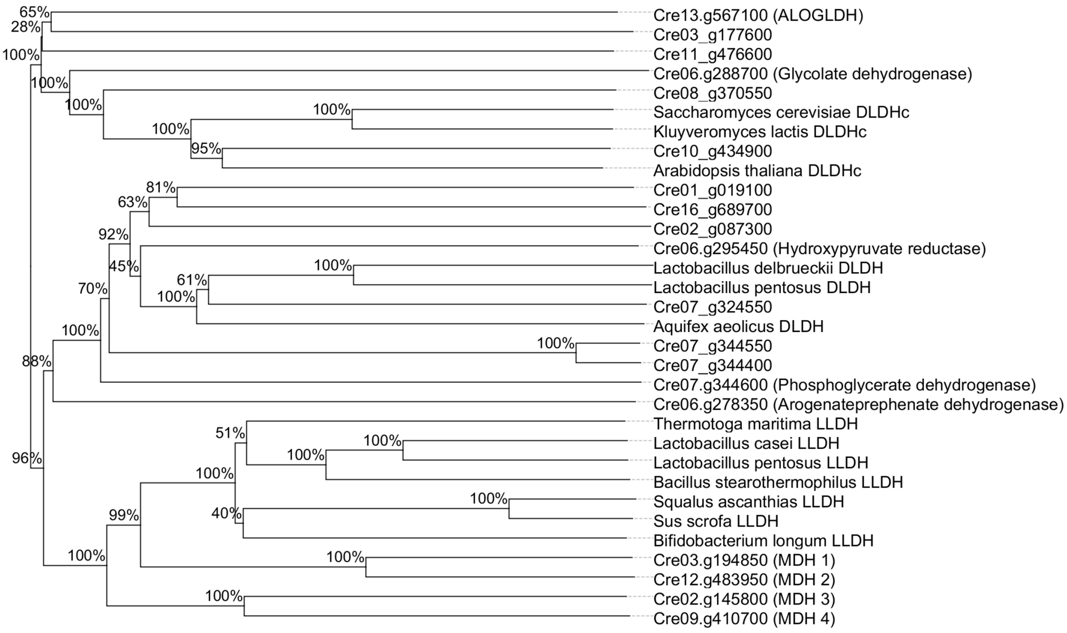

Figure S6: Phylogenetic analysis of LDHs. The Phytozome database was searched for potential LDHs using known LDHs i.e. NAD-dependent D- and L-LDH (labelled as DLDH and LLDH, respectively) and cytochrome-dependent D-LDH (labelled as DLDHc). The following sequences are included: *Aquifex aeolicus* (NP_213499.1), *Lactobacillus pentosus* (D-LDH: P26298.1; L-LDH: P56511.1), *L. delbrueckii* (P26297.3), *Sus scrofa* (NP_001165834.1), *Squalus acanthias* (AAA91038.1), *Bifidobacterium longum* (AAA22900.1), *Thermotoga maritima* (1a5z), *L. casei* (P00343.3), *Bacillus stearothermophilus* (P00344.1), *Kluyveromyces lactis* (CAA50635.1), *Saccharomyces cerevisiae* (NP_010107.1) and *Arabidopsis thaliana* (AED91037). Protein accessions are given according to the NCBI database except for *T. maritima* (RCSB Protein Data Bank) and *C. reinhardtii* (Phytozome). Abbreviations: MDH, malate dehydrogenase; ALOGLDH, arabinono lactone oxidase/galactono-lactone dehydrogenase.
