# Supplementary material for: Identification of the Elusive Pyruvate Reductase of Chlamydomonas reinhardtii Chloroplasts
Source: Plant Cell Physiol. 2015 Nov 15;57(1):82–94. doi: 10.1093/pcp/pcv167 (PMC4722173; doi:10.1093/pcp/pcv167)
Supplement: Supplementary Data [file supp_pcv167_suppl_data.zip › pcp-2015-e-00308-File020.pdf]

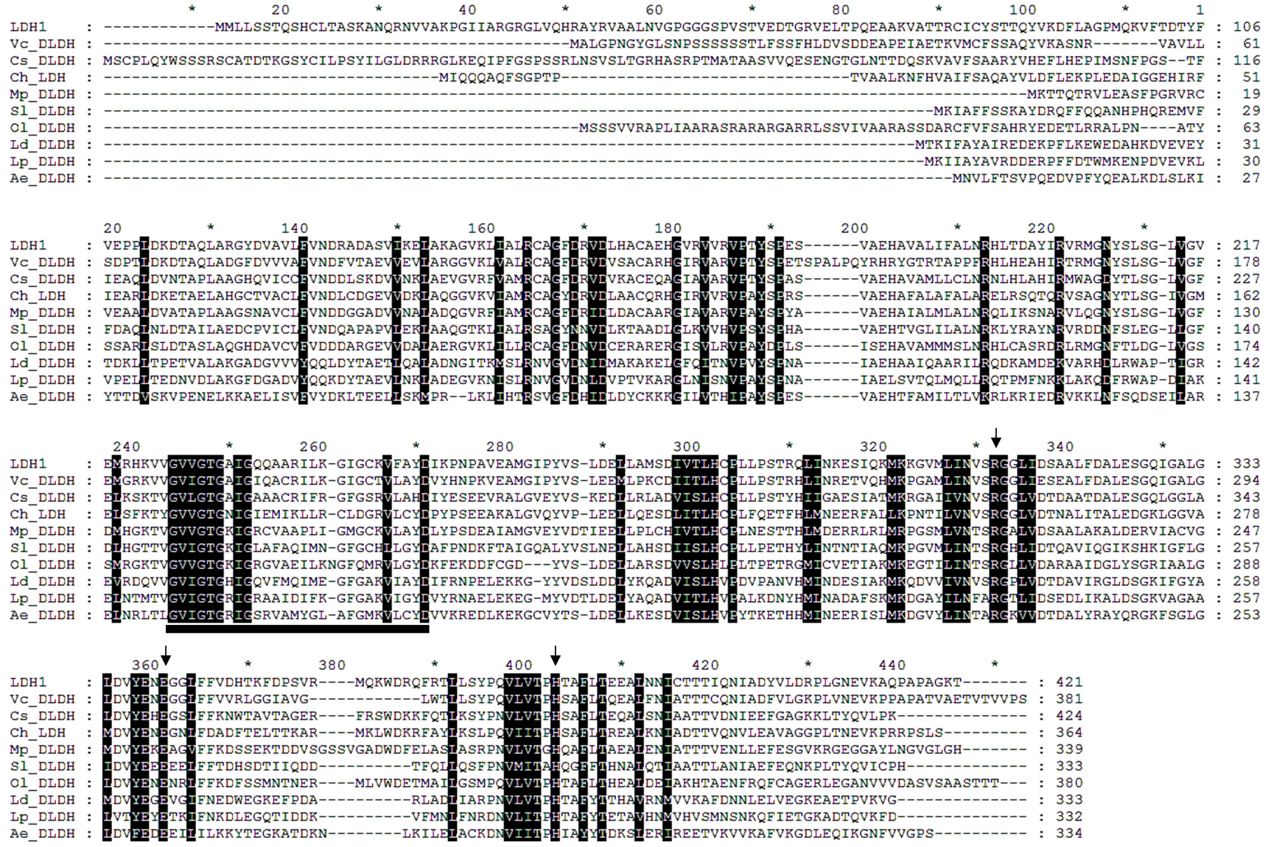

Figure S7: Protein sequence alignment of putative *Chlamydomonas reinhardtii* NAD dependent D-LDH (Phyotozome ID: Cre07.g324550), with NAD dependent D-LDHs. Aquifex aeolicus VF5 (NP_213499.1), *Chlorella* NC54A (133093) *Coccomyxa* sp. C-1693 (28806a), *Micromonas pusilla* CCMP1545 (147892)*, Lactobacillus delbrueckii* (P26297.3), *Lactobacillus pentosus* (P26298.1), *Ostreococcus lucimarinus* (37878), *Synechocystis* sp. PCC 6803 (slr1556), *Volvox carteri f. nagariensis* (Vocar20012819m). Active site residues are indicated by black arrows (Lapierre et al. 1999) and the NAD binding motif (GXXGXGXXGX17D) is underlined. Protein accessions are given according to Phytozome v10 or NCBI database.
