# Supplementary material for: Identification of the Elusive Pyruvate Reductase of Chlamydomonas reinhardtii Chloroplasts
Source: Plant Cell Physiol. 2015 Nov 15;57(1):82–94. doi: 10.1093/pcp/pcv167 (PMC4722173; doi:10.1093/pcp/pcv167)
Supplement: Supplementary Data [file supp_pcv167_suppl_data.zip › pcp-2015-e-00308-File021.pdf]

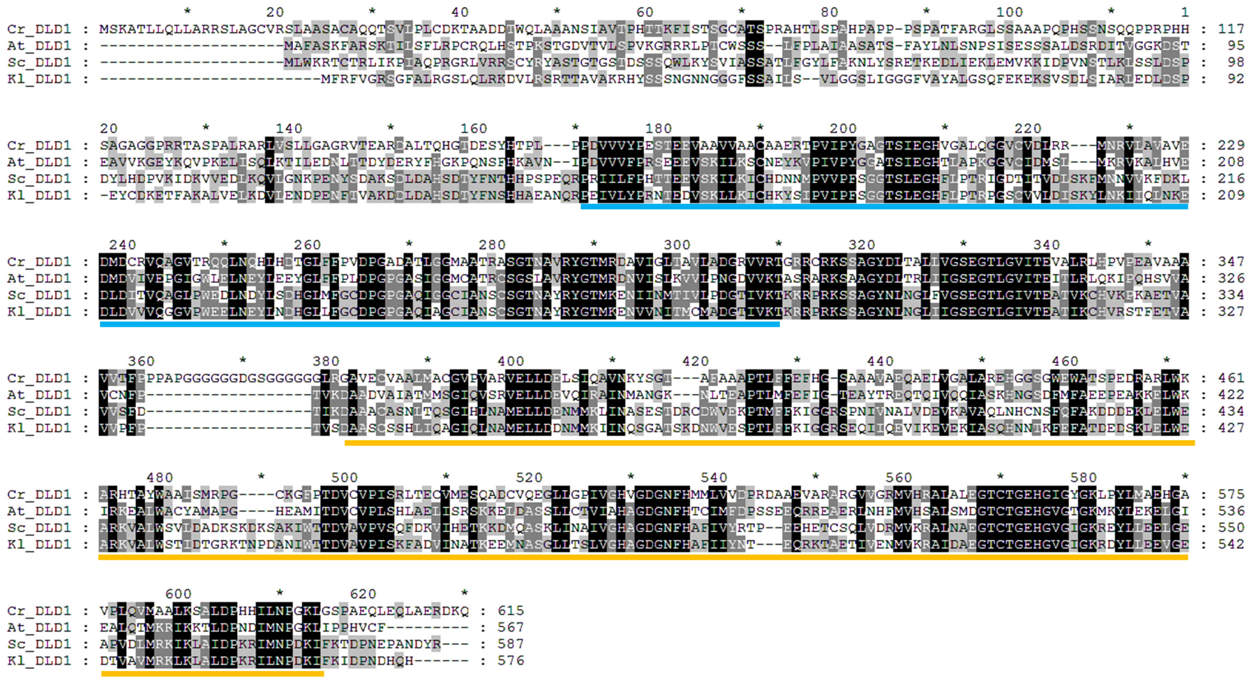

Figure S8: Protein sequence alignment of putative *C. reinhardtii* FAD dependent LDH (CrDLD1, Phytozome ID: Cre10.g434900), with sequences from *Arabidopsis thaliana* (AT5G06580), *Kluyveromyces lactis* (Q12627) and *Saccharomyces cerevisiae* (YDL174C). Functional domains are highlighted: FAD binding 4 (light blue, pfam ID: PF01565); FAD-oxidase C (yellow, pfam ID: PF02913).
