# Supplementary material for: Identification of the Elusive Pyruvate Reductase of Chlamydomonas reinhardtii Chloroplasts
Source: Plant Cell Physiol. 2015 Nov 15;57(1):82–94. doi: 10.1093/pcp/pcv167 (PMC4722173; doi:10.1093/pcp/pcv167)
Supplement: Supplementary Data [file supp_pcv167_suppl_data.zip › pcp-2015-e-00308-File022.pdf]

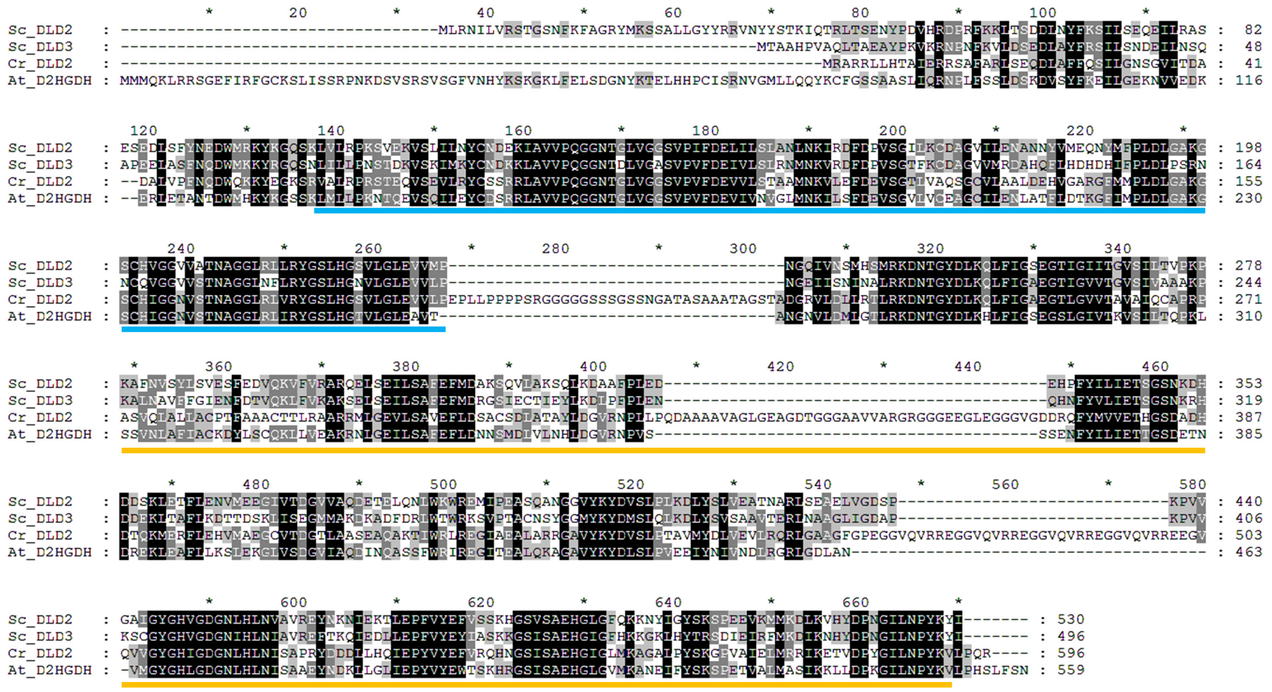

Figure S9: Protein sequence alignment of putative *C. reinhardtii* FAD dependent LDH (CrDLD2, Phytozome ID: Cre08.g370550), with sequences from *Arabidopsis thaliana* (At_D2HGDH, AT4G36400), *Saccharomyces cerevisiae* (DLD2, YDL178W) and *Saccharomyces cerevisiae* (DLD3, YEL071W). Functional domains are highlighted: FAD binding 4 (light blue, pfam ID: PF01565); FAD-oxidase C (yellow, pfam ID: PF02913).
