# Supplementary material for: Identification of the Elusive Pyruvate Reductase of Chlamydomonas reinhardtii Chloroplasts
Source: Plant Cell Physiol. 2015 Nov 15;57(1):82–94. doi: 10.1093/pcp/pcv167 (PMC4722173; doi:10.1093/pcp/pcv167)
Supplement: Supplementary Data [file supp_pcv167_suppl_data.zip › pcp-2015-e-00308-File023.pdf]

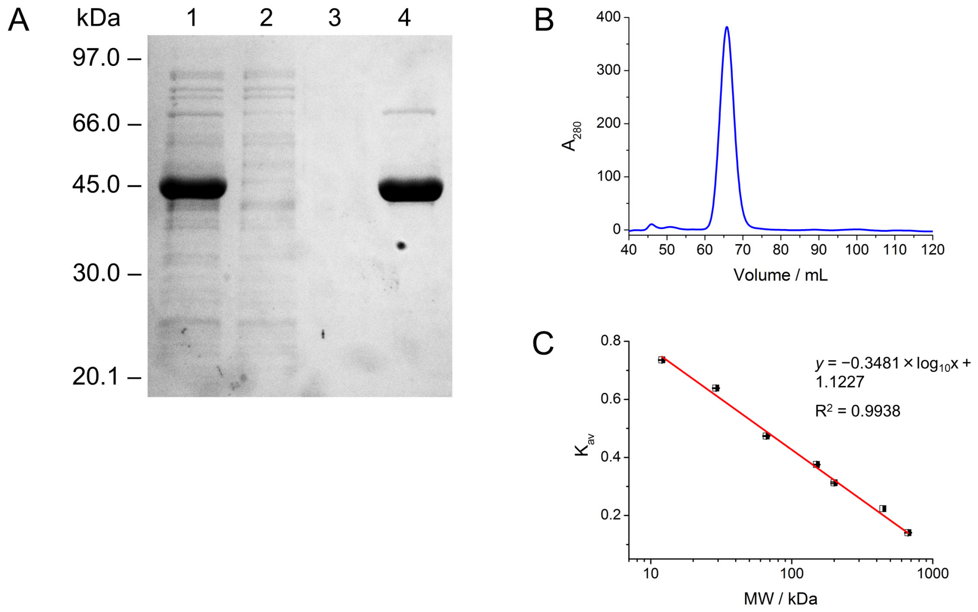

Figure S10: (A) Immobilised metal ion affinity purification of recombinant *Cr*-LDH1. Lane 1: *E. coli* cell lysate soluble fraction; lane 2: flow-through; lane 3: wash; lane 4: elution. (B) Gel filtration elution profile of recombinant *Cr*-LDH1 under native conditions. A single intense protein peak eluted at 65.8 mL. (C) Gel filtration protein standard curve used to estimate the molecular weight of the native *Cr*-LDH1 oligomer. Seven protein standards (Sigma-Aldrich, Gillingham, UK) were used: cytochrome c (12.4 kDa), carbonic anhydrase from bovine erythrocytes (29 kDa), BSA (66 kDa), yeast alcohol dehydrogenase (150 kDa), β-amylase from sweet potato (200 kDa), apoferritin from horse spleen (443 kDa), and bovine thyroglobulin (669 kDa). The gel phase distribution coefficient (K_{av}) was recorded as a function of molecular weight (MW), where $K_{av} = (V_e - V_o)/(V_t - V_o)$, V_e is the protein elution volume, V_o is the void volume and V_t is the total volume of the column. The data were plotted on a semi-log plot and fit with a linear function. The MW of the native *Cr*-LDH1 oligomer was estimated to be 200 kDa based on the elution volume from (B).
