# Supplementary material for: Identification of the Elusive Pyruvate Reductase of Chlamydomonas reinhardtii Chloroplasts
Source: Plant Cell Physiol. 2015 Nov 15;57(1):82–94. doi: 10.1093/pcp/pcv167 (PMC4722173; doi:10.1093/pcp/pcv167)
Supplement: Supplementary Data [file pcp-2015-e-00308-File016.pdf › pcp-2015-e-00308-File016.pdf]

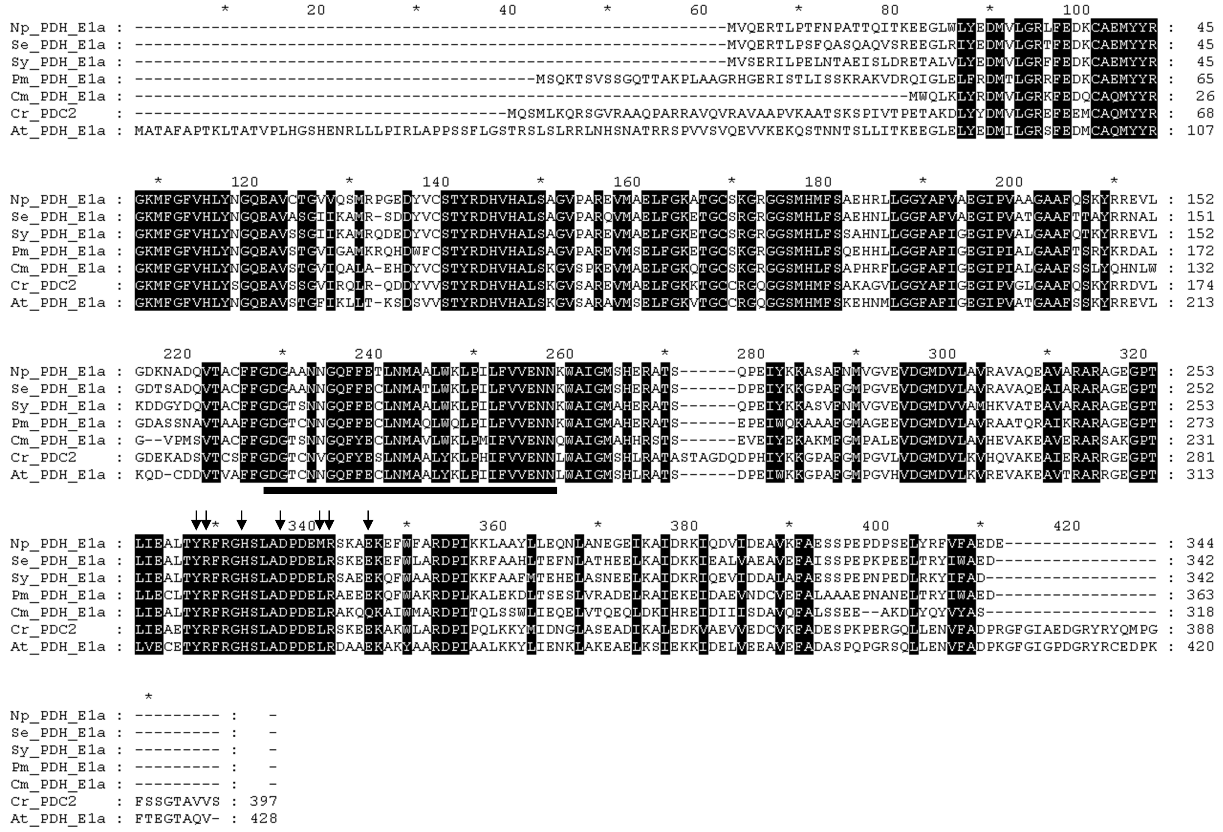

Figure S3: Protein sequence alignment comparing *Chlamydomonas reinhardtii* PDC2 (Phytozome ID: Cre02.g099850) with putative plant chloroplast and cyanobacteria pyruvate dehydrogenase E1 alpha components. *Cyanidioschyzon merolae* (NP_849059), *Arabidopsis thaliana* (NP_171617.1), *Nostoc punctiforme* (YP_001868828), *Synechocystis* PCC6803 (NP_441914), *Prochlorococcus marinus* (YP_001017966.1). Active site residues are indicated by black arrows (Fries et al. 2003) and the TPP binding motif GDG-X24-27-NN is underlined (Hawkins et al. 1989). Protein accessions given according to NCBI database (http://www.ncbi.nlm.nih.gov/protein).
