# Supplementary material for: Identification of the Elusive Pyruvate Reductase of Chlamydomonas reinhardtii Chloroplasts
Source: Plant Cell Physiol. 2015 Nov 15;57(1):82–94. doi: 10.1093/pcp/pcv167 (PMC4722173; doi:10.1093/pcp/pcv167)
Supplement: Supplementary Data [file supp_pcv167_suppl_data.zip › pcp-2015-e-00308-File011.doc]

**Table S1:** Putative mitochondrial PDH complex components

| **#** | **Alias** | **Phytozome ID** | **Chlre v4 Protein ID** | **Predicted Location** | **IUBMB** |
| --- | --- | --- | --- | --- | --- |
| E1α | PDC1 | Cre07.g337650 | 193810 | C/M | EC 1.2.4.1 |
| E1β | PDH1a | Cre01.g063700 | 139515 | –/M | EC 1.2.4.1 |
| E2 | DLA1 | Cre29.g778200 | 149709 | C/M | EC 2.3.1.12 |
| E2 | DLA3 | Cre06.g252550 | 187285 | –/M | EC 2.3.1.12 |
| E3 | DLD1 | Cre31.g780600 | – | –/M | EC 1.8.1.4 |

Subcellular location predictions are displayed with results from ChloroP/TargetP

**Table S2:** Putative chloroplast PDH complex components

| **#** | **Alias** | **Phytozome ID** | **Chlre v4 Protein ID** | **Predicted Location** | **IUBMB** |
| --- | --- | --- | --- | --- | --- |
| E1α | PDC2 | Cre02.g099850 | 155587 | C/– | EC 1.2.4.1 |
| E1β | PDH2 | Cre03.g194200 | 190446 | C/M | EC 1.2.4.1 |
| E2 | DLA2 | Cre03.g158900 | 196500 | C/– | EC 2.3.1.12 |
| E3 | DLD2 | Cre01.g016500 | 205763 | C/C | EC 1.8.1.4 |

Subcellular location predictions are displayed with results from ChloroP/TargetP

**Table S3:** Crystallographic data collection and refinement statistics for Cr-LDH1

| **Crystal parameters** | |  |
| --- | --- | --- |
|  | |  |
| Space group | | P 2_1_ |
| Cell dimensions | | a=83.75 Å b=110.92 Å  c=193.160 Å  α=90.00º β=90.11º γ=90.00º |
| **Data collection** | |  |
|  | |  |
| Beamline | | Diamond Light Source I02 |
| Wavelength (Å) | | 0.97950 |
| Resolution (Å) | | 66.84–2.46 (2.52–2.46) |
| Unique observations | | 126364 (9378) |
| R_merge_ | | 0.061 (0.367) |
| Mean (<*I*> / σ*I*) | | 8.4 (1.8) |
| Completeness (%) | | 98.7 (99.2) |
| Multiplicity | | 2.6 (2.6) |
|  | |  |
| **Refinement** | |  |
| R_work_ / R_free_ (%) | | 20.2 / 25.4 |
| Number of protein residues | | 2745 |
| RMSD stereochemistry | |  |
|  | Bond lengths (Å) | 0.011 |
|  | Bond angles (°) | 1.5 |
| Ramachandran analysis (%) | |  |
|  | Residues in outlier regions | 4 |
|  | Residues in favoured regions | 2664 |
| Numbers in parentheses refer to the outermost resolution shell.  R_merge_ = Σ \|*I* – <*I*>\| / Σ *I* where *I* is the integrated intensity of a given reflection and <*I*> is the mean intensity of multiple corresponding symmetry-related reflections.  R_work_ = Σ \|\|F_o_\| – \|F_c_\|\| / Σ F_o_ where F_o_ and F_c_ are the observed and calculated structure factors respectively.  R_free_ = R_work_ calculated using ~5% random data excluded from the refinement.  RMSD stereochemistry is the deviation from ideal values. | | |
